# From Diarylsulfides to Diarylamines: New Ebola Virus Entry Inhibitors with Improved Metabolic Stability

**DOI:** 10.1021/acs.jmedchem.5c00615

**Published:** 2025-05-17

**Authors:** Marcos Morales-Tenorio, Fátima Lasala, Alfonso Garcia-Rubia, Elnaz Aledavood, Michelle Heung, Catherine Olal, Beatriz Escudero-Pérez, Paola Oquist, Ángeles Canales, Covadonga Alonso, Ana Martínez, César Muñoz-Fontela, Rafael Delgado, Carmen Gil

**Affiliations:** † 54446 Centro de Investigaciones Biológicas Margarita Salas (CIB-CSIC), 28040 Madrid, Spain; ‡ Instituto de Investigación Hospital 12 de Octubre, 28041 Madrid, Spain; § Bernhard Nocht Institute for Tropical Medicine, 20359 Hamburg, Germany; ∥ Facultad de Ciencias Químicas, 16734 Universidad Complutense de Madrid, 28040 Madrid, Spain; ⊥ Dpt. Biotechnology, Instituto Nacional de Investigación y Tecnología Agraria y Alimentaria (INIA-CSIC), 28040 Madrid, Spain; # CIBERNED, Instituto Salud Carlos III, 28029 Madrid, Spain; ∇ CIBERINFEC, Instituto Salud Carlos III, 28029 Madrid, Spain; ○ School of Medicine, 16734 Universidad Complutense de Madrid, 28040 Madrid, Spain

## Abstract

The persistence of current outbreaks of Ebola virus (EBOV) disease and challenges in the production and administration of approved vaccines and treatments highlight the continuous exploration of new therapeutic alternatives. In this context, this work focuses on optimizing diarylsulfide hits previously identified as EBOV entry inhibitors. Structural modifications resulted in diarylamine derivatives, with confirmed antiviral activity against replicative EBOV and significantly improved metabolic stability compared to diarylsulfides. Using different techniques, the EBOV glycoprotein (EBOV-GP) was identified as the target of these compounds. Residue Y517_GP2_ is critical for biological activity, while T519_GP2_, E100_GP1_, and D522_GP2_ also contribute to ligand binding. Furthermore, the binding of the derivatives to EBOV-GP has been shown to destabilize the complex with the virus receptor NPC1. In short, a new family of diarylsulfides and diarylamines with antiviral activity against EBOV has been developed, and their mechanism of action has been deciphered, paving the way for future pharmaceutical development.

## Graphical abstract

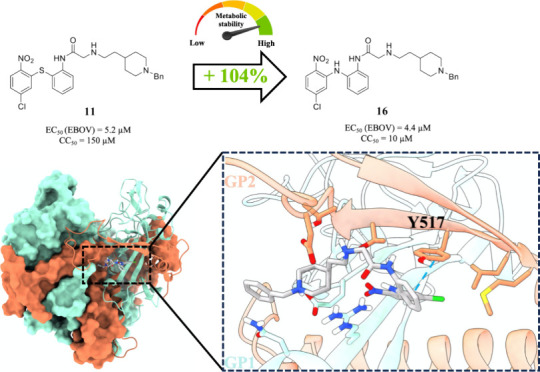

## Introduction

Ebola virus (EBOV) has become in last years a major public health pathogen due to its high mortality and the limited therapeutic options available to date for treating Ebola virus disease (EVD). The 2013–2016 epidemic in West Africa with more than 11,000 deaths raised international concern and effort to explore new ways to combat the virus if new outbreaks were to occur.^1^ This international cooperation bore fruit at the end of 2020 when the first treatments to combat EVD were approved. The first vaccine for EBOV based on the recombinant vesicular stomatitis virus (rVSV) with the Ebola virus glycoprotein (EBOV-GP) in its envelope and, two monoclonal antibodies treatments were approved by the FDA.^2–4^ However, outbreaks continue to occur sporadically and unpredictably in Africa with the most recent one caused by Sudan virus (Orthoebolavirus sudanense, SUDV), which is still ongoing in Uganda at the time of writing.^5^ This situation highlights the ongoing challenges faced by the African population to have access to treatments. Moreover, the production of monoclonal antibodies as a treatment for the disease presents a major industrial challenge due to the high cost and difficulty of the process. The treatment of EVD would greatly benefit with an effective and globally accessible treatment easy to demand to target population.^6^ This is the reason why the search for small molecule antiviral candidates remains a primary goal in the development of anti-Ebola drugs.^7^ In order to optimize the identification of new molecules active against the virus it is essential to understand how the Ebola virus infects cells. EBOV belongs to the *Filoviridae* family in the order *Mononegavirales*.^8^ Virions consist of a set of viral proteins (VP24, VP30, VP35 and VP40), an RNA-dependent polymerase (L), a nucleoprotein (NP), two soluble glycoproteins (sGP and ssGP) and a virion-attached glycoprotein (EBOV-GP). EBOV-GP is the key for viral entry and consists of two subunits, GP1 and GP2, which assembled into a 450-kDa trimer at the surface of nascent virions.^9,10^ EBOV-GP is protected by a glycan cap that must be removed in order to infect cells. Infection process starts with Ebola virus entry to the cells via macropinocytosis by the attachment of EBOV to β-integrins and to C-type lectins such as DC-SIGN and L-SIGN on the cell surface.^11^ Once the virus is internalized it is transported from early endosomes to late endosomes, where cathepsins B and L produce the cleavage of the glycan cap of Ebola virus glycoprotein (EBOV-GPcl),^12^ necessary for the exposure of GP1 to the Niemann-Pick C1 receptor (NPC1), a cholesterol transporter that acts as the natural receptor of this virus.^13,14^ Once EBOV-GP1 and the domain C of NPC1 (NPC1-C) interacts, EBOV-GP2 mediates the membrane fusion process and the release of the viral genome occurs. The key role of NPC1/EBOV-GP interaction in the viral entry process, has sparked interest in disrupting this interaction as an appealing strategy for developing antivirals that act as virus entry inhibitors.^15^ In terms of reported compounds with anti-EBOV activity acting at this level, there are a number of drugs approved for other indications that target a hydrophobic pocket located between the two subunits of the virus glycoprotein, GP1 and GP2, first identified for toremifene.^16–18^ This binding destabilizes the EBOV-GPcl/NPC1-C complex, thereby preventing viral infection.

In a previous work, we performed a virtual screening using the crystallographic structure of the EBOV-GPcl/NPC1-C complex^19^ and our in-house chemical library (MBC library),^20^ finding two interesting chemical families of small molecules active in an assay with EBOV-GP-pseudotyped viruses.^21^ The one having a carbazole core, was optimized leading to a novel series of *N*′-phenylacetohydrazide derivatives with confirmed antiviral activity in replicative EBOV. These novel antivirals interfere with the NPC1/EBOV-GP interaction by binding to the GP1 and GP2 interface, particularly targeting Y517, a critical residue for drug interaction, as seen with toremifene.^22^ Following this path, we present here the optimization of the second family of hits, sulfides **SC073** and **SC198** (Figure 1). Structural modifications of diarysulfides were focused on the improvement of the synthetic accessibility together with improving antiviral and pharmacokinetic properties. Moreover, experimental validation of the proposed mechanism of action through disruption of NPC1/EBOV-GP interaction is presented herein, based on Saturation Transfer Difference–Nuclear Magnetic Resonance (STD–NMR) and site-directed mutagenesis studies. A detailed computational analysis of this mechanism is also included.

**Figure 1:**
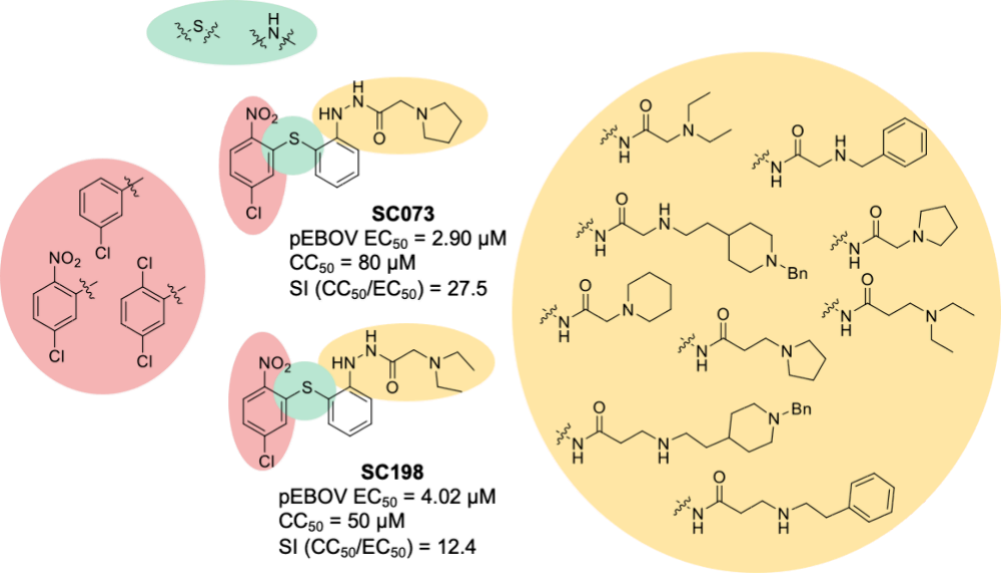
Structural optimization of sulfide derivative hits, **SC073** and **SC198**, as EBOV entry inhibitors.

## Results and Discussion

### Chemistry

Our two hits, diarylsulfides **SC073** and **SC198**, comprise two phenyl rings linked by a sulfur atom. One of the aromatic rings has a nitro group at the *ortho* position and a chlorine at the *meta* position relative to the sulfur. The other phenyl ring has a hydrazide moiety linked to the ring through the nitrogen at *ortho* position with respect to the sulfur and a chain with an aliphatic amine attached to the carbonyl group.

Structural modifications of diarysulfides (Figure 1) primarily involved the replacement of the lateral hydrazide with new chains of varying lengths, wherein the hydrazide was replaced by an amide group. This substitution was motivated by the easier chemical synthesis of amides. Additionally, the central sulfur atom was replaced by amine to enhance the metabolic stability of the final compounds, as sulfides are prone to oxidation during metabolism.^23^

The synthetic preparation of diarylsulfide and diarylamine derivatives is shown in Scheme 1. For sulfides **7**-**15**, 2,4-dichloro-1-nitrobenzene and 2-aminothiophenol were used as starting materials to obtain sulfide **1** following a previously described procedure.^24^ The next step was the amide formation between the free amine group of sulfide **1** and the corresponding acyl chloride, 2-chloroacetyl or 3-chloropropionyl chloride to obtain sulfides **3** and **4** respectively. The final step consisted of the nucleophilic substitution of the chlorine from **3** and **4** with different amines in basic medium. This lead to diarylsulfides **7**-**15** (Table 1) in which terminal amines such as pyrrolidine, diethylamine, piperidine, benzylamine, phenethylamine or 2-(1-benzylpiperidin-4yl)­ethan-1-amine were included (Scheme 1).

**Scheme 1:**
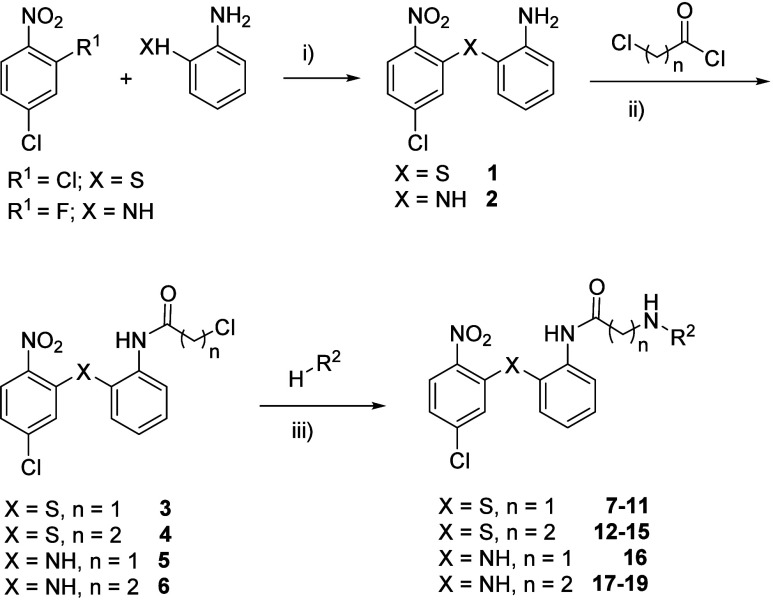
Synthesis of Diarylsulfide (**7**-**15**) and Diarylamine (**16**-**19**) Derivatives ^a^

a (i) K_2_CO_3_, CH_2_Cl_2_, 80 °C MW, 3 h when X = SH and DMSO, 150 °C MW, 3 h when X = NH; (ii) (Et_3_N), CH_2_Cl_2_, rt, 24 h; (iii) Et_3_N or DIPEA, ACN, 80 °C, 24 h.

**Table 1:**
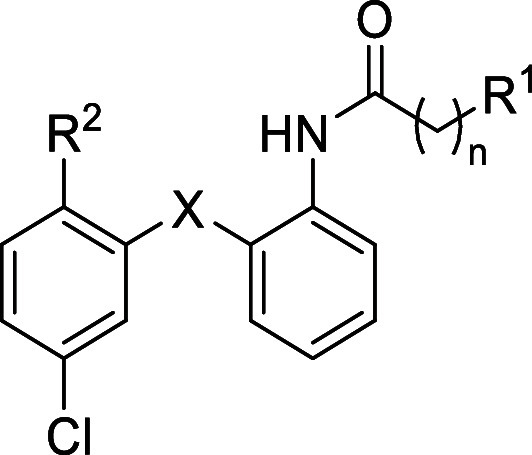

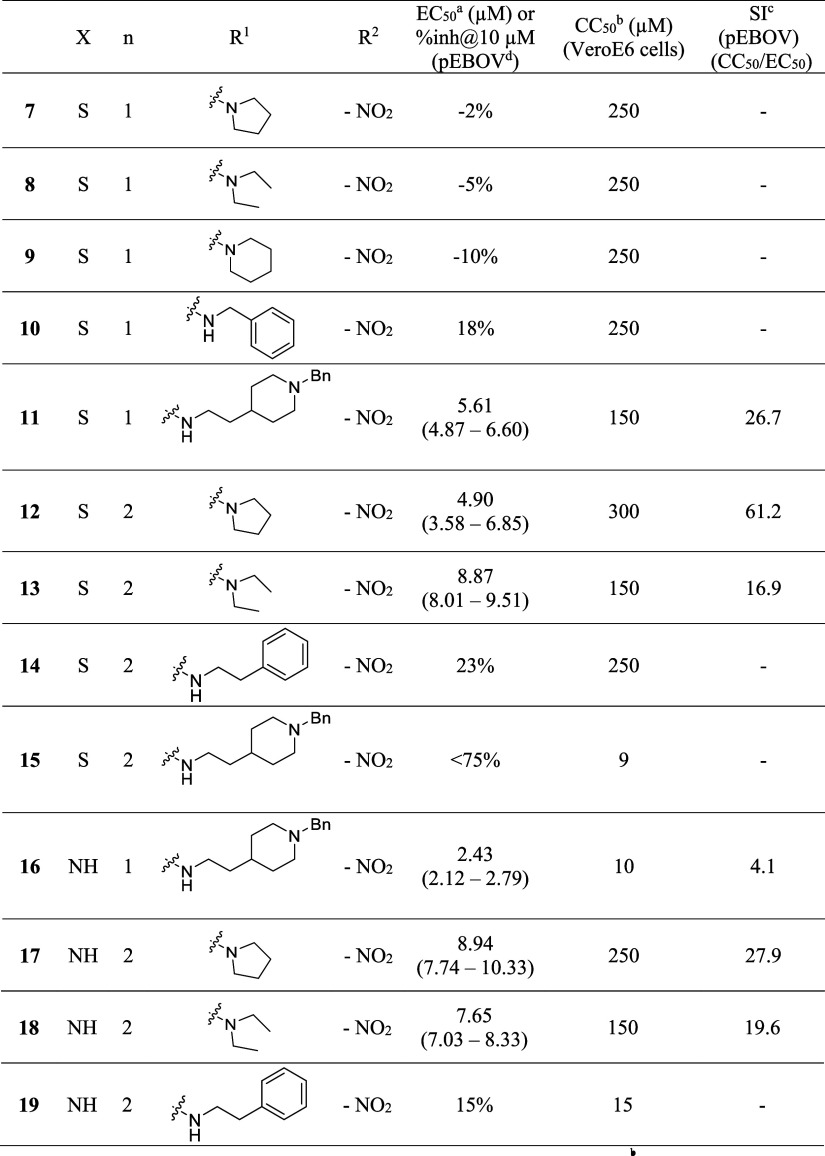
Antiviral Activity of the New Derivatives against EBOV-GP-pseudotype Virus (pEBOV)

a EC_50_: 50% effective concentration (with 95% confidence intervals in parentheses).

b CC_50_: 50% cytotoxic concentration.

c SI (CC_50_/EC_50_): Selectivity index.

d Toremifene was used as the reference of the assay EC_50_ = 0.17 μM (95% CI = 0.15–0.54 μM), CC_50_ (VeroE6 cells) = 10 μM, SI = 58.8 (lit^25^: EC_50_ = 0.07 ± 0.05 μM, CC_50_ (A549 cells) = 16 μM, SI = 229).

As mentioned above, compounds in which sulfur is replaced by amine were also synthesized to increase metabolic stability. In this sense, some diarylamine derivatives were prepared following a similar procedure described in Scheme 1. To obtain diarylamines **16**-**19**, 4-chloro-2-fluoro-1-nitrobenzene reacted with 1,2-diaminobenzene to yield diamine **2**. Subsequent reaction with 2-chloroacetyl or 3-chloropropionyl chloride yielded amides **5** and **6** respectively. Finally, diarylamines **16**-**19** were obtained with 2-(1-benzylpiperidin-4yl)­ethan-1-amine, pyrrolidine, diethylamine and phenetylamine as terminal amines (Scheme 1, Table 1).

### Screening against Pseudotype Model of EBOV

The antiviral potential of diarylsulfide and diarylamine derivatives here synthesized were initially evaluated in a pseudotype model of EBOV (pEBOV) in the same way as hits **SC073** and **SC198**. VeroE6 cells were infected with viral pseudotypes of human vesicular stomatitis virus (VSV) expressing EBOV-GP on their surface. Toremifene was used as reference compound in the assay showing an EC_50_ of 0.17 μM (95% CI = 0.15–0.54 μM). Previously reported antiviral data in A549 cells infected with human immunodeficiency virus (HIV) pseudotypes bearing the EBOV-GP showed an EC_50_ of 0.07 ± 0.05 μM.^25^ The slight discrepancy between these values may be attributed to differences in the pseudotype systems and host cells used. Overall, the results are consistent and support the reliability of the assay.

As observed in Table 1, diarylsulfides **7** and **8**, analogs of the two hits in which the hydrazide was replaced by an amide bond, were found inactive. However, diarylsulfides **12** and **13** which maintain the same terminal amines as the hits (pyrrolidine and diethylamine respectively) but have a lateral chain with one more carbon, show activity in the same range as the hits. This observation can be explained by the fact that in the case of diarylsulfides **12** and **13**, the addition of one more methylene result in a lateral chain with the same length as the hits. Remarkably, diarylsulfide **11**, with 2-(1-benzylpiperidin-4yl)­ethan-1-amine as a lateral chain, similar to ones present in other families of antiviral compounds,^22^ also exhibited activity in the micromolar range. The rest of sulfides evaluated (**9**, **10**, **14** and **15**) showed no activity in the antiviral assay. Comparing selectivity indexes (SI), sulfide **12** has a better SI than hit **SC073** (61.2 *vs* 27.5), whereas sulfide **13** has a slightly improved SI compared to hit **SC198** (16.9 *vs* 12.4).

Diarylamines **16**, **17**, **18** and **19** analogs of diarylsulfides **11**, **12**, **13** and **14** respectively were evaluated for comparative purposes. Derivative **19** was found inactive as its analog **14**, while the other diarylamines showed similar activities than the parent sulfides: **16** *vs* **11** (2.43 μM *vs* 5.61 μM), **17** *vs* **12** (8.94 μM *vs* 4.90 μM) and **18** *vs* **13** (7.65 μM *vs* 8.87 μM). In terms of selectivity index, diarylsulfides **11** and **12** exhibited a superiority over diarylamines **16** and **17**, while **13** and **18** are similar.

As control of selectivity, antiviral activity of selected compound with cells infected with viral pseudotypes with the vesicular stomatitis virus envelope GP (VSV-G) was determined. Remarkably, none of the tested compounds showed antiviral activity in this system, highlighting their specificity for targeting EBOV-GP (Table S1 of the Supporting Information).

### Confirmation Assays against Infectious EBOV

To validate our results, we checked the activity of the initial hits (**SC073** and **SC198**), a number of top candidates (**11**–**13** and **16**–**18**), and the inactive sulfide **7** in infected VeroE6 cells with the wild-type EBOV (Orthoebolavirus zairense, Mayinga variant). The assay’s performance was previously validated using favipiravir as a known EBOV inhibitor.^26^ This serves as a positive control and confirms the reliability of the assay.

As observed in Table 2, the inactive derivative **7** in the pseudotype assay was also found inactive in the assay with EBOV. Moreover, the two hits, **SC073** and **SC198**, together with active compounds **11** and **16**–**18**, showed antiviral activity also against the full replicant EBOV. A big discrepancy occurs with sulfide **12**, which is active against the surrogate system although inactive against EBOV. Also, a significant loss of activity in the assay with the replicative virus was found with sulfide **13** and diarylamines. These differences could be due to the different infectious systems used in both assays.

**Table 2:** Antiviral Activity of Selected Compounds against Replicative EBOV

	EC_50_ ^a^ (μM)	CC_50_ ^c^ (μM)	SI^d^
	(EBOV^b^)	(VeroE6 cells)	(CC_50_/EC_50_)
**SC073**	4.30	80	18.6
	(3.12–5.69)		
**SC198**	6.00	50	8.3
	(5.33–6.77)		
**7**	inactive		
**11**	5.20	150	28.8
	(4.43–6.05)		
**12**	inactive	300	
**13**	44.52	150	3.3
	(12.71–155.91)		
**16**	4.42	10	2.3
	(3.77–5.17)		
**17**	6.48	250	38.5
	(3.61–10.40)		
**18**	28.67	150	5.2
	(20.21–40.65)		

a EC_50_: 50% effective concentration (with 95% confidence intervals in parentheses).

b Favipiravir was used as reference of the assay (EC_50_ = 67 μM (95% CI = 56–75 μM), CC_50_ > 1000 μM, SI = 14.9).^26^

c CC_50_: 50% cytotoxic concentration.

d SI (CC_50_/EC_50_): Selectivity index.

In general terms, we found a nice correlation between the antiviral data in the pseudotype model and in the replicative virus, with diarylamine **17** being the compound with better therapeutic window.

### Metabolic Stability

One important factor to take into account in preclinical phases of drug development is the metabolic stability of compounds. This factor provides information on how long the administered compound remains in the organism in its original chemical form before it is metabolized by the organism. Given the expected poor metabolic stability of the sulfide here reported,^23^ the replacement of the sulfur atom with a nitrogen was performed, resulting in diarylamine derivatives. The metabolic stability of these derivatives was then assessed alongside the corresponding sulfides. The assay was performed using hepatic microsomes of both mouse and human, showing a significant increase in the half-life observed for diarylamine **16**, **17**, and **18** over diarylsulfide **11**, **12**, and **13** derivatives (Table 3).

**Table 3:** In Vitro Microsomal Stability of Tested Compounds in Liver Microsomes of Different Species

	metabolic stability in human liver microsomes		metabolic stability in mouse liver microsomes	
Compd.	*t*_1/2_ (min)	CL_int_ ^a^ (mL/min/mg protein)	*t*_1/2_ (min)	CL_int_ ^a^ (mL/min/mg protein)
**11**	23 ± 4	25 ± 4	6 ± 1	390 ± 70
**12**	46 ± 2	12.5 ± 0.6	10 ± 3	260 ± 80
**13**	16 ± 3	36 ± 8	6.4 ± 0.9	390 ± 60
**16**	47 ± 7	12 ± 2	8 ± 1	310 ± 50
**17**	136.2 ± 0.5	4.19 ± 0.02	20 ± 3	130 ± 17
**18**	63 ± 5	9.1 ± 0.7	14 ± 6	180 ± 70
**verapamil**	22 ± 2	26 ± 3	10 ± 1	250 ± 30

a CL_int_, intrinsic clearance.^27^

In particular, modification of diarylsulfide **13** to diarylamine **18** resulted in an increase in metabolic stability of 294% in human microsomes. The modification from diarylsulfide **11** to diarylamine **16** led to an increase of 105%, and from diarylsulfide **12** to diarylamine **17**, an increase of 195%. In the case of mouse microsomes, working with lower values, the increase in metabolic stability observed is smaller.

The observed increase in the metabolic stability of diarylamines may result from the reduced susceptibility of the nitrogen atom to oxidation compared to the sulfur atom, which leads to more metabolically stable compounds.

### STD-NMR Experiments

According to our previous results obtained in the development of the family of *N*′-phenylacetohydrazide derivatives where we found that these compounds interfered with the NPC1/EBOV-GP interaction through their binding at the GP1 and GP2 interface, thereby preventing viral infection,^22^ we proposed that the new family of diarylamines developed here could act in the same manner. In this way, we studied the binding of one of the compounds with lower EC_50_, diarylamine **16**, to EBOV-GP protein. For this purpose, we employed saturation transfer difference–nuclear magnetic resonance (STD–NMR) experiments which can be used to identify protein–ligand interactions.^28^ Acquiring the STD spectra of **16** in the presence of EBOV-GP, as shown in Figure 2, clear STD signals were found, indicating that the diarylamine **16** binds to the protein. The STD spectrum shows signals corresponding to the aromatic region of the compound, indicating that these regions are in contact with EBOV-GP (Figure 2A).

**Figure 2:**
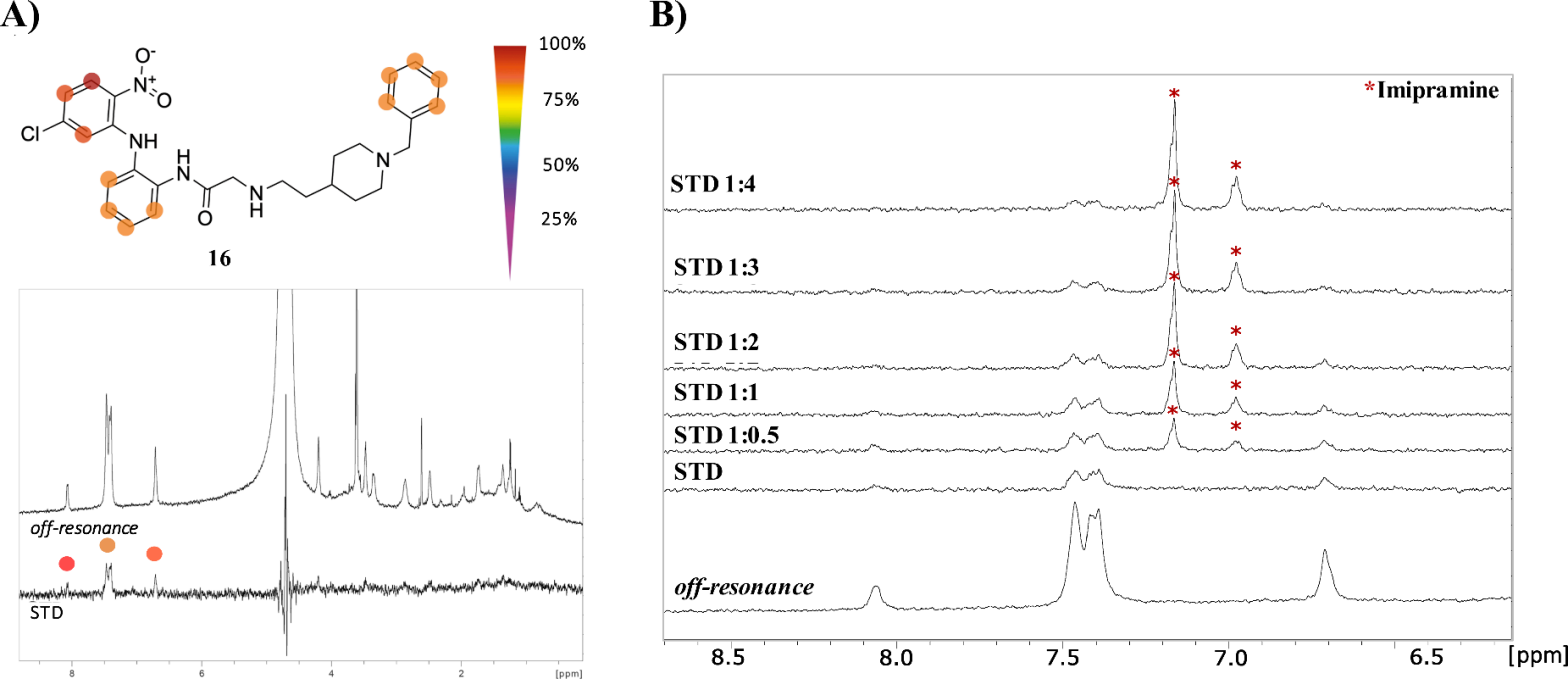
(A) Off-resonance and STD spectrum of **16** in the presence of EBOV-GP protein. The signals with higher STD effects were labeled with circles. (B) Competition experiment with increasing concentrations of imipramine. Off-resonance and STD spectra of **16** are shown as a reference (bottom spectra). Upon addition of imipramine, STD signals of **16** decrease, and imipramine STD signals increase. Imipramine signals are labeled with asterisks.

In order to confirm the binding of diarylamine **16** to the pocket located in the GP1 and GP2 interface, we performed a competition assay with imipramine as we have already described in our previous work.^22^ Imipramine is an antidepressant with antiviral activity against EBOV which is crystallized in this hydrophobic pocket of EBOV-GP.^18^ Addition of imipramine to the complex formed between **16** and EBOV-GP caused a decrease in the STD signal intensity of **16** which was confirmed with the addition of higher concentrations of imipramine (Figure 2B), indicating that both compounds target the same binding site of EBOV-GP.

### Computational Modeling of Inhibitor Binding

As noted in the introduction, the interaction between small molecules and the internal fusion loop region of the EBOV-GP has been identified as a mechanism that inhibits the virus from entering host cells.^16^ The GP is a homotrimer formed by heterodimers, consisting of two subunits: GP1 and GP2. GP1 is responsible for the initial attachment to the host cell surface, while GP2 is involved in the fusion of the viral membrane and the release of viral ribonucleic acid (RNA) into the host cell cytoplasm.^29^ Consequently, small molecules that can bind to the hydrophobic groove at the GP1-GP2 interface could allosterically modify the interaction of the GP complex with the NPC1 protein.^22^

To thoroughly understand the binding mode and inhibitory mechanism of this new chemical class of viral entry inhibitors, we employed docking and molecular dynamics (MD) simulations. For this study, the crystal structure of the EBOV-GP in complex with toremifene with protein data bank identifier (PDB ID): 5JQ7 ^16^ was used to dock the diarylamine derivative **16**. The binding mode with the highest docking score, −8.446, was subsequently subjected to MD simulations.

In the MD simulated model for compound **16**, the 5-chloro-2-nitrophenyl group is positioned within the hydrophobic pocket near the L515 and M548 residues, forming a π–π stacking interaction with the Y517 residue of GP2, which is one of the crucial interactions between inhibitors and the GP protein, as highlighted by mutagenesis studies.^30^ Additionally, the two protonated amines extend into a pocket region containing polar or charged residues such as R64, N61, E100, T519, T520, and D522, which engage in direct hydrogen-bonding or salt-bridge interactions with the ligand (Figure 3).

**Figure 3:**
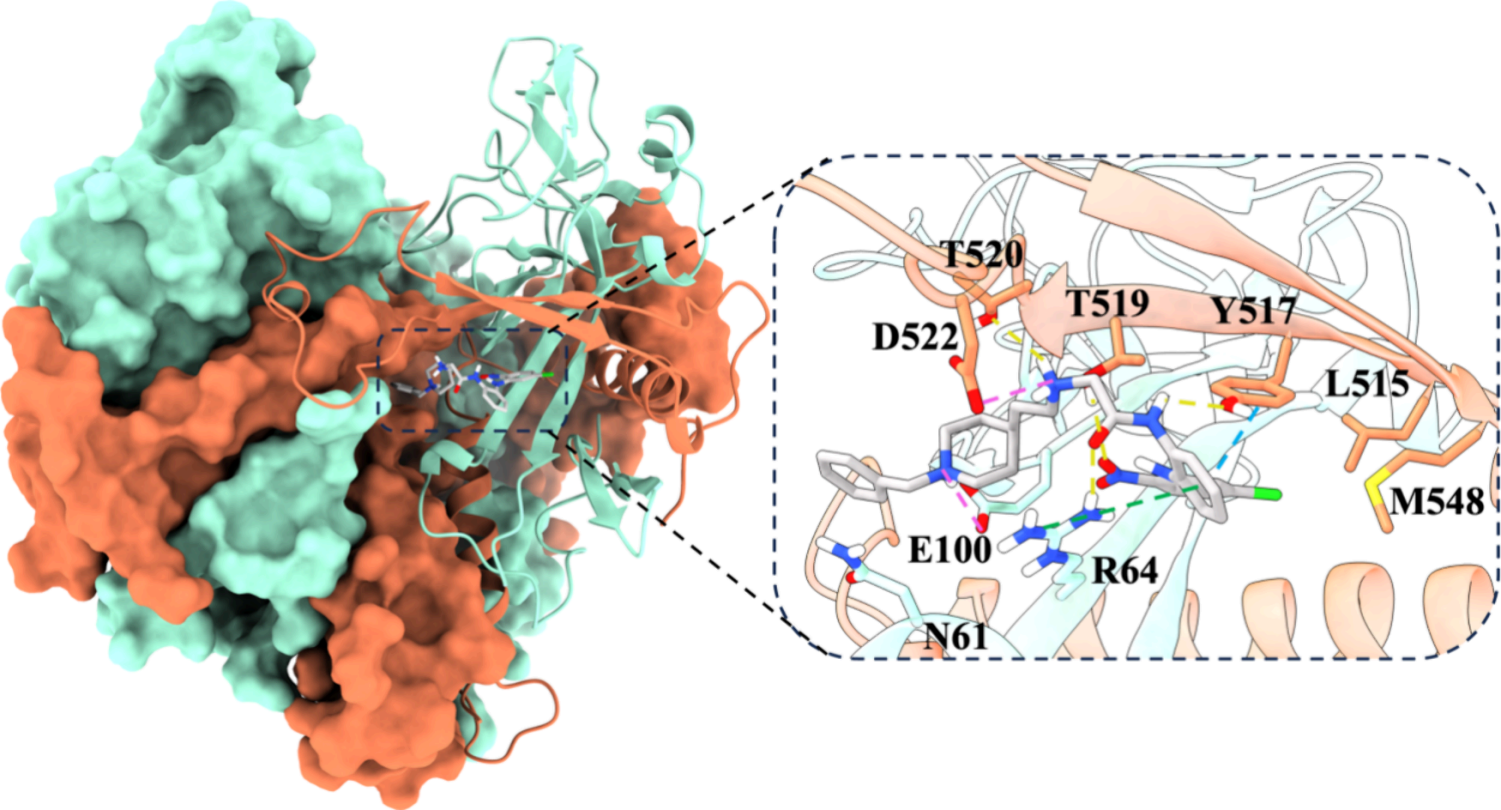
Representative binding mode of compound **16** (rendered in gray sticks) within the cavity, formed between GP1 (illustrated as a cyan cartoon) and GP2 (depicted as a salmon cartoon), as identified from 1.5 μs of MD simulations. Detailed close-up of the ligand binding pocket, highlighting essential residues within a 5Å proximity of the ligand. Other heterodimers of GP1-GP2 are depicted with a protein surface. The yellow dashed lines denote hydrogen bonds, while the pink dashed lines indicate salt bridges, the blue dashed line represents π–π stacking and green dashed line shows π-cation interaction. The X-ray structure of the EBOV-GP in complex with toremifene (PDB ID: 5JQ7) was used as a starting point for molecular modeling studies.

In comparison to compound **16**, the binding mode of **17**, evaluated after 1.5 μs of MD simulations, is illustrated in Figure S1 in the Supporting Information. The 5-chloro-2-nitrophenyl group occupies the hydrophobic subcavity that was filled by the same group in compound **16**, and it engages in π–π stacking interactions with Y517_GP2_. Additionally, the positively charged amine groups can form salt bridge with D522_GP2_ in the fusion loop. The reduced activity observed for compound **17** may be partly attributed to fewer protein–ligand interactions and suboptimal coverage of the binding site by this compound. As a result, this lack of interaction is anticipated to adversely affect the binding mode’s overall stability.

The binding stability and the impact of the compound **16** on GP’s structure were assessed by analyzing the positional root-mean-square deviation (RMSD) and root-mean-square fluctuation (RMSF) of the protein backbone during simulations. The RMSD analysis for the protein backbone atoms (shown in the black profile in Figure 4) and the ligand (depicted in violet profile in Figure 4) demonstrated the strong stability of compound **16** throughout the 500 ns MD simulations in three replicas. Additionally, the RMSF profile reveals that GP1 experiences the greatest fluctuations in residues 110–120 near the receptor binding site (RBS), while GP2 exhibits significant fluctuations in residues 520–540 within the fusion loop (Figure S2 from the Supporting Information).

**Figure 4:**
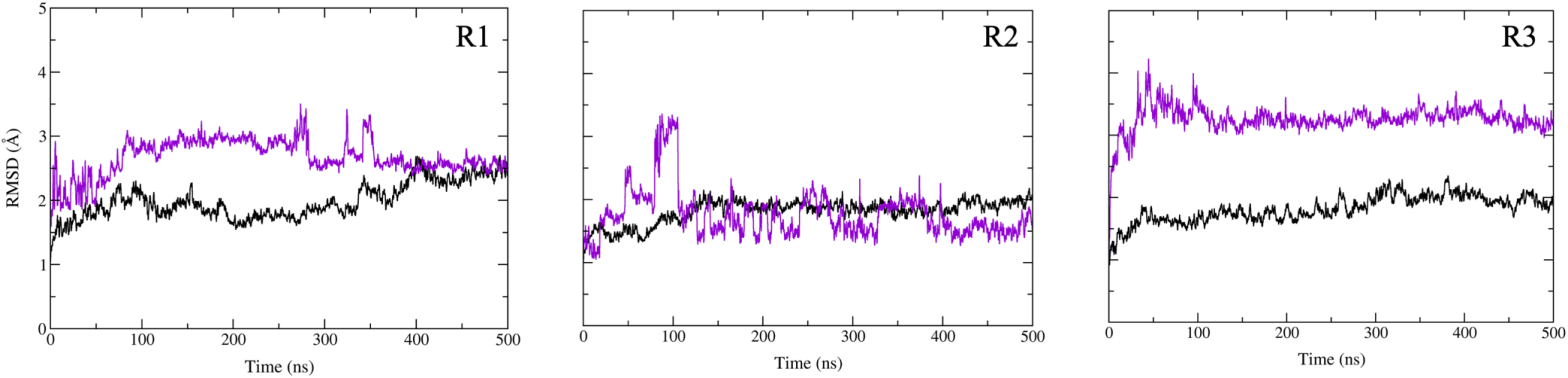
RMSD values (in Å) over the 500 ns of simulations for three independent replicas of GP-**16** complexes with protein backbone atoms indicated in black and the ligand highlighted in violet.

The impact of the inhibitor’s binding on the dynamics of GP was further investigated using essential dynamics (ED) analysis. ED was employed to comprehend how the binding of the ligand influences the main motions of the protein backbone. The analysis results from ED unveiled that the binding of the inhibitor triggers alterations in the conformation of the GP complexes, resulting in shifts in both the receptor-binding site (RBS) region and the fusion loop (Figure 5B). These conformational changes augment the flexibility in these critical areas essential for NPC1 binding, which is significant for the virus’s ability to infect cells. Conversely, in the absence of the inhibitor in the apo system, structural motions in the aforementioned regions are notably tempered, especially regarding the fusion loop and the RBS which are less flexible compared to the ligand-bound state (Figure 5A).

**Figure 5:**
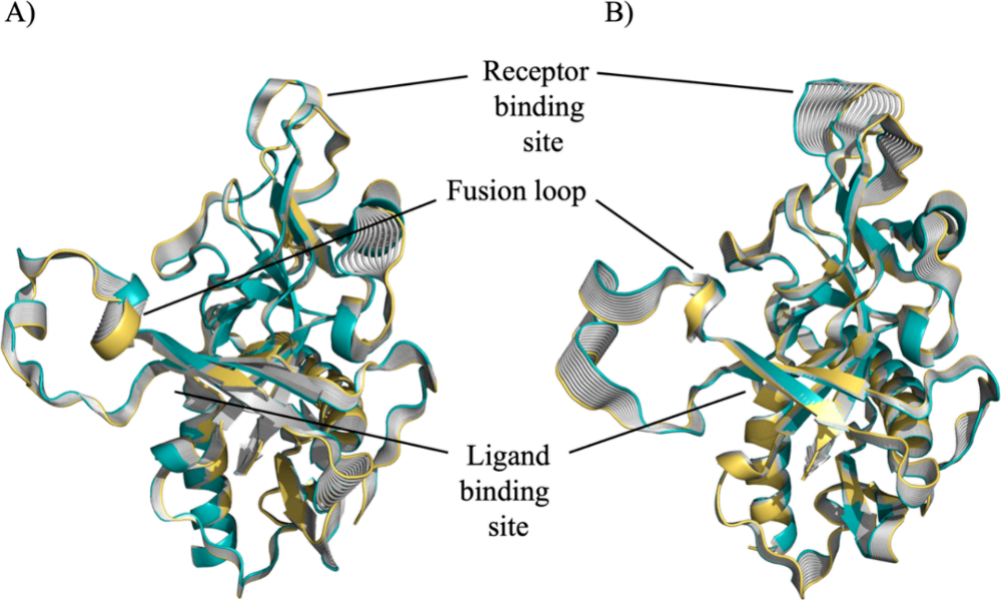
Representation of the first essential motion derived from the ED analysis of the protein backbone for (A) apo and (B) GP-**16** complex determined from the snapshots sampled along the 500 ns of MD simulations. The X-ray structure of the EBOV-GP in complex with toremifene (PDB ID: 5JQ7) was used as a starting point for molecular modeling studies.

The increased flexibility of the protein arising upon binding of the inhibitor to the fusion loop can be viewed as a mechanism to translate the binding energy into changes in the protein dynamics that should inhibit the engagement of GP with NPC1 and entering the virus to the host cell.

To understand how NPC1 binding inhibition is achieved through compound **16** binding in the fusion loop, we conducted an interaction network analysis using the weighted implementation of suboptimal paths (WISP)^31^ tool. This tool helps identify protein residues that may work together to connect two distinct binding sites. Our results for both the apo (unbound) and holo (ligand-bound) states revealed networks of residues linking the fusion loop and receptor binding site. A comparison of these states (Figure 6) shows a significant increase in the number of GP1 residues involved in these networks when the inhibitor is present, along with an increase in the number of paths connecting the ligand binding site to the receptor binding site (thick blue lines in Figure 6B). In the apo state, the fusion loop (D522) and RBS (D117) are connected by a single pathway (shown in blue in Figure 6A), involving Y542_GP2_, T175_GP1_ and E120_GP1_, indicating a weaker connection compared to the holo state. In the holo state, the interaction network becomes more complex, suggesting increase in the protein flexibility. Besides the initial pathway seen in the apo state, two additional pathways are formed involving correlated movements through Y542_GP2_-A125_GP1_-R172_GP1_ and Y542_GP2_-F132_GP1_-I112_GP1_-P116_GP1_ (Figure 6B). Thus, compound **16** enhances protein flexibility and the connections between the fusion loop (allosteric binding site) and the RBS, thereby inhibiting GP attachment to the receptor.

**Figure 6:**
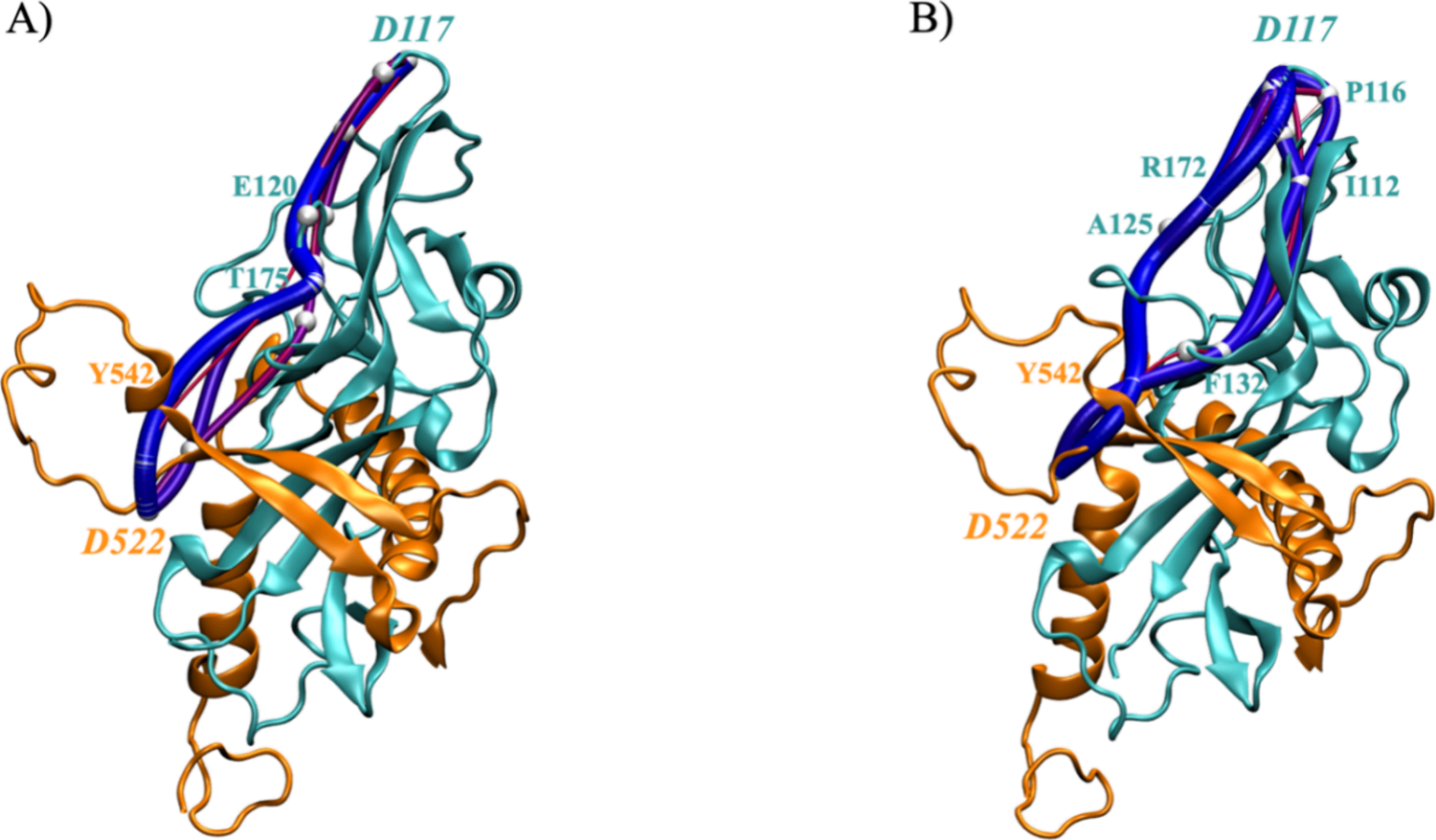
Representation of the optimal pathways indicating the residue interaction network between the fusion loop and the RBS for the simulations of (A) apo and (B) holo. The wide blue lines indicate the shortest pathways, and thinner red and violet lines display the longest pathways. GP1 and GP2 are depicted as cyan and orange cartoons, respectively. The key residues involved in the connection path are represented as white nodes. The X-ray structure of the EBOV-GP in complex with toremifene (PDB ID: 5JQ7) was used as a starting point for molecular modeling studies.

### Experimental Confirmation of the Mechanism of Action

Based on the results from the STD experiment and the in silico analysis of allosteric inhibition in the GP-NPC1 complex, derivative **16** appears to exert its antiviral action by binding to a hydrophobic pocket located at the interface between the GP1 and GP2 subunits of the EBOV-GP. To support these findings, we proposed the experimental confirmation of this binding by mutating the Y517 residue of EBOV-GP.^22,30^ Y517 is a critical residue for stabilizing drug binding in this pocket, where other drugs like toremifene or imipramine also binds.^16,18^ The EBOV-GP Y517S mutation led to a drastic loss of potency for diarylamine **16**, showing similar results to those obtained with the reference inhibitors toremifene and imipramine when compared to pseudoviruses expressing wild type EBOV-GP (Figure 7A). As observed in the computational study, the binding of derivative **16** to this pocket is likely to enhance the flexibility of the protein and the connections between the fusion loop and the RBS, thereby allosterically inhibiting EBOV-GP attachment to the NPC1 receptor. This was experimentally confirmed using an ELISA-based assay to study the effect of compound **16** on the binding of EBOV-GP to NPC1 domain C. As shown in Figure 7B, derivative **16** interferes with the interaction between NPC1 and EBOV-GP in a similar way to the positive control, imipramine.

**Figure 7:**
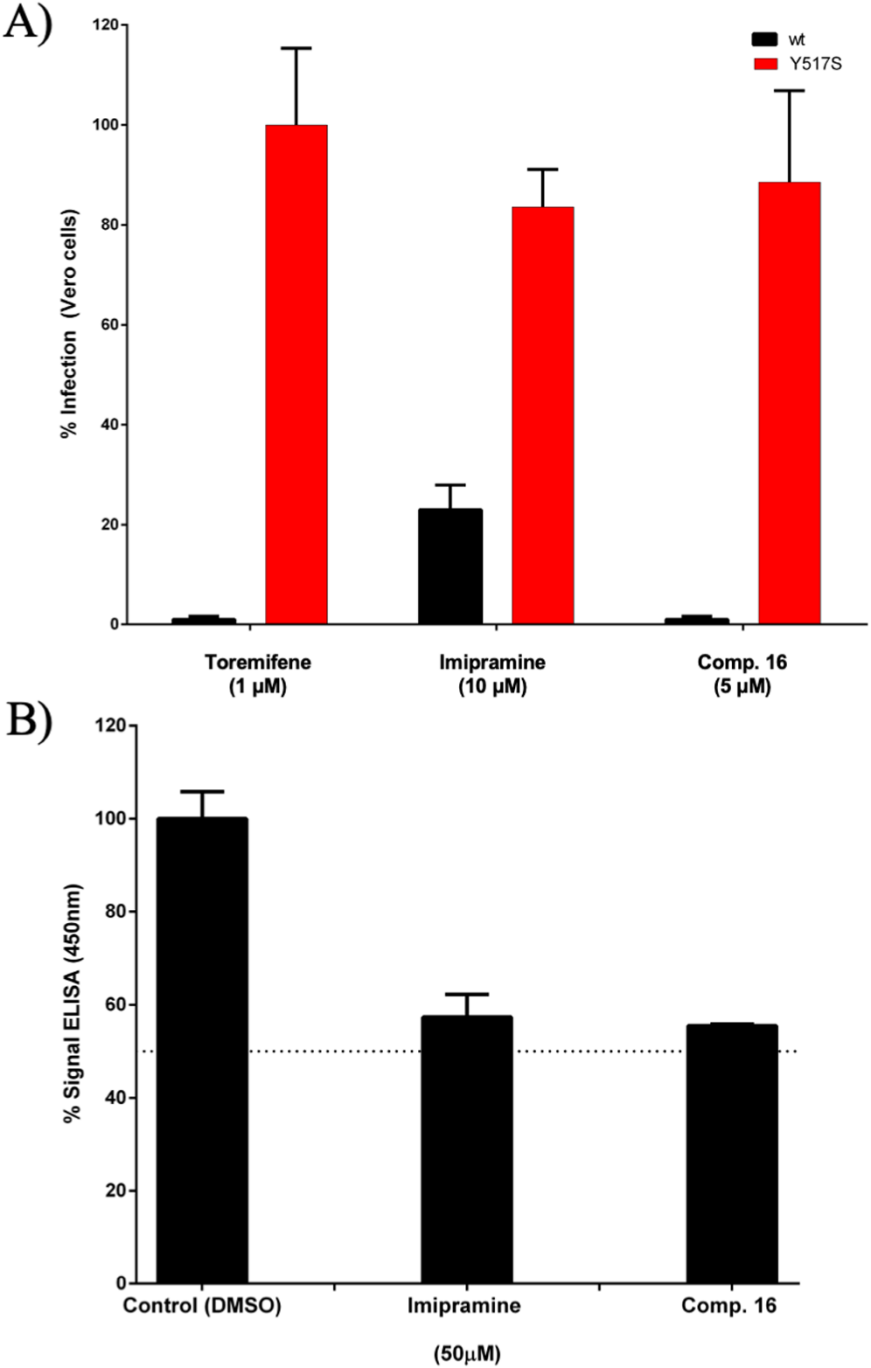
(A) Antiviral evaluation of diarylamine **16** (5 μM) against EBOV-GP Y517S mutant pseudotyped virus in Vero cells infected with either EBOV-GP wt or mutant pseudotyped rVSV. Following a 48-h incubation period, cells were lysed and examined for luciferase expression. The percentage of infection is depicted as 100% with toremifene for Y517S mutant. The concentration of tested compounds was selected based on the corresponding CC_50_. Error bars represent the standard deviation from three independent experiments. (B) Assessment of the inhibitory effect of selected diarylamine **16** on the interaction between EBOV-GPcl/NPC1-domain C by ELISA. ELISA plates, coated with cleaved EBOV particles, were subjected to incubation with hNPC1-domain C-flag in the presence or absence (control) of compound **16**, as well as positive control (imipramine). The bound domain C was subsequently detected utilizing an antiflag antibody conjugated to horseradish peroxidase, followed by Ultra-TMB substrate. Error bars represent the standard deviation from three independent experiments.

### Blood–Brain Barrier Permeability Prediction

The Ebola virus causes hemorrhagic fever in humans, often leading to death due to multiorgan failure. One organ commonly affected in survivors is the brain. While the exact mechanism by which EBOV impacts the central nervous system (CNS) is still unclear, neurological complications, including headaches, memory loss, and cranial nerve abnormalities, have been observed postinfection. This suggests that the virus can reach the brain, causing damage, and that treatments must be able to cross the blood-brain barrier to target the infection in all affected organs.^32^ Using a parallel artificial membrane permeability assay (PAMPA),^33^ we determined the predicted brain permeability for all the compounds with a promising activity against EBOV. In general, all the compounds evaluated present permeability values compatible with passive brain penetration, ensuring the arrival of the drug to the brain (Table 4).

**Table 4:** In Vitro Permeability (Pe) Values of the Derivatives Tested^a^

compound	Pe (10^–6^ cm·s^–1^)	prediction
**SC073**	11.4 ± 0.3	SNC +
**SC198**	11 ± 3	SNC +
**11**	6.2 ± 0.8	SNC +
**12**	16 ± 3	SNC +
**13**	14 ± 2	SNC +
**16**	9.7 ± 0.5	SNC +
**17**	15.9 ± 0.6	SNC +
**18**	18 ± 2	SNC +

a The results correspond to the mean ± standard deviation of two independent experiments.

## Conclusions

The currently available therapeutic options for treating Ebola virus disease (EVD) still present certain limitations, underscoring the urgent need for continued research into alternative treatments for a virus that continues to cause outbreaks in subsaharian Africa.

This study reports the discovery of a novel class of viral entry inhibitors based on a diarylsulfide scaffold, demonstrating activity against Ebola virus (EBOV). The initial diarylsulfide hits identified in the research, were subjected to structural modifications with the aim of enhancing their pharmacological properties. These modifications were also designed to establish structure–activity relationships (SAR), which would provide deeper insights into the molecules’ mechanisms of action.

In this context, not only additional diarylsulfide derivatives with anti-EBOV activity have been synthesized, but given the expected poor metabolic stability of these compounds, the replacement of the sulfur atom by a nitrogen atom was proposed, resulting in diarylamine derivatives with maintained activity and improved metabolic stability.

Moreover, through experimental techniques such as Saturation Transfer Difference Nuclear Magnetic Resonance (STD-NMR) and mutagenesis studies, the potential mechanism of action of these compounds has been elucidated. The compounds appear to inhibit viral entry by binding to a hydrophobic pocket at the interface between the two subunits of the EBOV glycoprotein (EBOV-GP), a hypothesis supported by comprehensive computational modeling.

In conclusion, this family of diarylsulfide and diarylamine derivatives holds significant promise for the development of novel therapeutic agents for the treatment of EVD.

## Experimental Section

### Chemistry

Analytical grade solvents purchased from Sigma-Aldrich were used for all reactions. Argon was used to perform the reactions in an inert atmosphere. Microwave reactions were carried out with an Initiator device (Biotage). Precoated aluminum foils (ALUGRAM Xtra SIL G/UV254, Merk) were used for thin layer chromatography to follow reactions. Melting points were recorded with a Büchi Melting point M-560 apparatus. ^1^H and ^13^C NMR spectra were recorded on a Bruker AV 300 MHz instrument (^1^H NMR, 300 MHz; ^13^C NMR, 75 MHz) or on a 500 MHz instrument (^1^H NMR, 500 MHz; ^13^C NMR, 125 MHz) located at the NMR unit of Research Assistance Centres from Complutense University of Madrid. The abbreviations used are s (singlet), d (doublet), t (triplet), q (quartet), m (multiplet). Coupling constants (*J*) are expressed in Hz. Mass spectra were acquired on a Thermo Mod. Finnigan LXQ spectrometer coupled to a high-performance liquid instrument equipped with ZORBAX SB-C18 column (50 mm × 4.6 mm, 3.5 μm packing diameter), using scan positive electrospray ionization (ESI). Column chromatography was performed on silica gel 60 (Merk) manually or automatically using the IsoleraOne instrument (Biotage). High resolution mass spectra (HRMS-ESI) were recorded on an Agilent 6500 mass spectrometer with ESI/APCI ionization source and quadrupole/time-of-flight (QTOF) coupled to an Agilent 1200 liquid chromatograph equipped with a Phenomenex Luna C18(2) reversed phase column (100 mm × 2.1 mm, 3 μm packing diameter) located at the Mass Spectrometry Service of the Institute of General Organic Chemistry (IQOG-CSIC). The HPLC conditions for purity assessment were as follows: HPLC Surveyor equipped with a PDA Surveyor plus UV–vis detector; ZORBAX SB-C18 column (3.5 μm, 4.6 mm × 50 mm); H_2_O/CH_3_CN gradient elution from 100/0 to 0/100 for 5, 7, or 10 min; flow rate 500 or 800 μL/min; wavelength, UV 254 nm. The following three different gradient conditions were used.

Gradient I: 23 °C, 0.5 mL/min flow rate. Gradient elution with the mobile phases as (A) H_2_O containing 0.1% volume/volume (v/v) formic acid and (B) CH_3_CN containing 0.1% (v/v) formic acid. Gradient conditions were initially 5% B, increasing linearly to 95% B over 5 min, remaining at 95% B for 1.45 min, and then decreasing to 10% B over 0.55 min.

Gradient II: 23 °C, 0.8 mL/min flow rate. Gradient elution with the mobile phases as (A) H_2_O containing 0.1% volume/volume (v/v) formic acid and (B) CH_3_CN containing 0.1% (v/v) formic acid. Gradient conditions were initially 5% B, increasing linearly to 100% B over 3 min, remaining at 100% B for 1.45 min, and then decreasing to 5% B over 0.55 min.

Gradient III: 23 °C, 0.8 mL/min flow rate. Gradient elution with the mobile phases as (A) H_2_O containing 0.1% volume/volume (v/v) formic acid and (B) CH_3_CN containing 0.1% (v/v) formic acid. Gradient conditions were initially 10% B, increasing linearly to 95% B over 5 min, remaining at 95% B for 4 min, and then decreasing to 0% B over 1 min.

All the final compounds are >95% pure by HPLC. Confirmation HPLC traces are included in the Supporting Information. Hit compounds, sulfides **SC073** and **SC198**, were synthesized following previously described procedures.^34^

#### 2-((5-Chloro-2-nitrophenyl)­thio)­aniline (**1**)^24^

2-Aminothiophenol (5.2 mmol, 556 μL) was added dropwise to a solution of 2,4-dichloro-1-nitrobenzene (5.2 mmol, 1 g) and K_2_CO_3_ (26 mmol, 3.6 g) in CH_2_Cl_2_ (12 mL). The mixture was heated under microwave irradiation for 180 min at 80 °C. Volatiles were evaporated to dryness under vacuum. The resulting residue was purified by flash column chromatography using a mixture of EtOAc/Hex (1:9). Yield: 435 mg (30%) as an orange solid. Mp 128–129 °C (lit.^24^ 134–135 °C). ^1^H NMR (300 MHz, DMSO-*d*_6_): δ 8.30 (d, *J* = 8.92 Hz, 1H), 7.44 (dd, *J* = 8.5, 2.2 Hz, 1H), 7.36 (dd, *J* = 7.6, 1.6 Hz, 1H), 7.30 (ddd, *J* = 8.3, 7.3, 1.5 Hz, 1H), 6.86 (dd, *J* = 8.2, 1.3 Hz, 1H), 6.68 (dd, *J* = 7.5, 1.3 Hz, 1H), 6.64 (d, *J* = 2.1 Hz, 1H), 5.65 (s, 2H).

#### *N* ^1^-(5-Chloro-2-nitrophenyl)­benzene-1,2-diamine (**2**)^35^

1,2-Diaminobenzene (6.27 mmol, 678 mg) and 4-chloro-2-fluoro-1-nitrobenzene (5.7 mmol, 1 g) were mixed in DMSO (10 mL). The mixture was heated under microwave irradiation for 180 min at 150 °C. The crude was washed with NaHCO_3_, H_2_O, saturated NaCl solution and the organic phase was extracted with EtOAc. The solvent was dried over anhydrous Mg_2_SO_4_. The desiccant was filtered off, the solvent was evaporated under reduced pressure and the resulting residue was purified by flash column chromatography using a mixture of EtOAc/Hex (1:9). Yield: 1.04 g (70%) as an orange solid. ^1^H NMR (300 MHz, CDCl_3_) δ 9.12 (s, 1H), 8.20–8.10 (m, 1H), 7.24–7.16 (m, 1H), 7.15–7.09 (m, 1H), 6.94–6.80 (m, 2H), 6.76–6.65 (m, 2H), 3.80 (s, 2H). ^13^C NMR (75 MHz, CDCl_3_) δ 144.9, 142.9, 131.4, 129.1, 128.4, 128.1, 123.1, 119.6, 117.9, 116.8, 115.5. HPLC-MS (gradient II/4 min) (M + H)^+^ = 264, *R*_t_ = 3.08 min (97%).

### General Procedure A for the Synthesis of **3**–**6**

The corresponding aniline (1 equiv) was dissolved in anhydrous CH_2_Cl_2_ and to this solution was added dropwise the acid chloride (1 or 1.2 equiv., indicated in each case) dissolved in anhydrous CH_2_Cl_2_. The reaction was stirred for 24 h at room temperature. At the end of the reaction time the CH_2_Cl_2_ was evaporated, the product was dissolved in EtOAc and the organic phase was washed with H_2_O and saturated NaCl solution and/or NaHCO_3_ solution and then dried over anhydrous Mg_2_SO_4_. The desiccant was filtered off, the volatiles were evaporated to dryness under vacuum and the resulting residue was purified by flash column chromatography using mixtures of solvents as eluents as indicated in each case.

#### 2-Chloro-*N*-(2-((5-chloro-2-nitrophenyl)­thio)­phenyl)­acetamide (**3**)

The title compound was prepared by reaction of 2-((5-chloro-2-nitrophenyl)­thio)­aniline (**1**) (1.37 mmol, 400 mg), 2-chloroacetyl chloride (1.6 mmol, 127 μL), Et_3_N (1.50 mmol, 209 μL), and CH_2_Cl_2_ as solvent (8 mL) according to procedure A. Compound **3** was obtained pure after workup. Yield: 320 mg (65%) as a brown solid. ^1^H NMR (300 MHz, DMSO-*d*_6_) δ 9.83 (s, 1H), 8.29 (d, *J* = 8.9 Hz, 1H), 7.87 (dd, *J* = 8.1, 1.4 Hz, 1H), 7.73 (dd, *J* = 7.7, 1.5 Hz, 1H), 7.70–7.62 (m, 1H), 7.47 (dd, *J* = 8.8, 2.2 Hz, 1H), 7.41 (td, *J* = 7.6, 1.4 Hz, 1H), 6.56 (d, *J* = 2.1 Hz, 1H), 4.16 (s, 2H).

#### 3-Chloro-*N*-(2-((5-chloro-2-nitrophenyl)­thio)­phenyl)­propanamide (**4**)

The title compound was prepared by reaction of 2-((5-chloro-2-nitrophenyl)­thio)­aniline (**1**) (1.6 mmol, 435 mg), 3-chloropropionyl chloride (1.9 mmol, 178 μL) and CH_2_Cl_2_ as solvent (8 mL) according to procedure A. Compound **4** was obtained pure after workup. Yield: 520 mg (91%) as a brown solid. ^1^H NMR (300 MHz, DMSO-*d*_6_) δ 9.65 (s, 1H), 8.26 (d, *J* = 8.8 Hz, 1H), 7.89–7.81 (m, 1H), 7.69 (dd, *J* = 7.7, 1.4 Hz, 1H), 7.67–7.58 (m, 1H), 7.43 (dd, *J* = 8.8, 2.2 Hz, 1H), 7.40–7.33 (m, 1H), 6.54 (d, *J* = 2.2 Hz, 1H), 3.70 (t, *J* = 6.2 Hz, 2H), 2.68 (t, *J* = 6.2 Hz, 2H). ^13^C NMR (75 MHz, DMSO-*d*_6_) δ 168.6, 143.5, 140.5, 139.5, 139.2, 137.1, 131.8, 127.7, 126.8, 126.6, 126.4, 125.9, 122.5, 40.5, 38.4.

#### 2-Chloro-*N*-(2-((5-chloro-2-nitrophenyl)­amino)­phenyl)­acetamide (**5**)

The title compound was prepared by reaction of *N* ^1^-(5-chloro-2-nitrophenyl)­benzene-1,2-diamine (**2**) (0.97 mmol, 256 mg), 2-chloroacetyl chloride (0.97 mmol, 76 μL) and CH_2_Cl_2_ as solvent (6 mL) according to procedure A. Compound **5** was obtained pure after workup. Yield: 330 mg (99%) as a yellow solid. ^1^H NMR (300 MHz, CDCl_3_) δ 9.21 (s, 1H), 8.58 (s, 1H), 8.26–8.15 (m, 2H), 7.46–7.38 (m, 1H), 7.37–7.27 (m, 2H), 6.80 (dd, *J* = 9.1, 2.2 Hz, 1H), 6.69 (d, *J* = 2.1 Hz, 1H), 4.14 (s, 2H). ^13^C NMR (75 MHz, CDCl_3_) δ 164.2, 144.1, 143.0, 133.6, 132.2, 128.8, 128.6, 128.3, 127.8, 126.6, 122.7, 118.9, 115.4, 43.1. HPLC-MS (gradient II) (M + H)^+^ = 340, *R*_t_ = 3.61 min (98%).

#### 3-Chloro-*N*-(2-((5-chloro-2-nitrophenyl)­amino)­phenyl)­propanamide (**6**)

The title compound was prepared by reaction of *N* ^1^-(5-chloro-2-nitrophenyl)­benzene-1,2-diamine (**2**) (2.9 mmol, 763 mg), 3-chloropropionyl chloride (2.9 mmol, 277 μL) and CH_2_Cl_2_ as solvent (12 mL) according to procedure A. Compound **6** was obtained pure after workup. Yield: 1.02 g (99%) as a yellow solid. ^1^H NMR (300 MHz, CDCl_3_) δ 9.18 (s, 1H), 8.23–8.13 (m, 2H), 7.61 (s, 1H), 7.43–7.35 (m, 1H), 7.33–7.22 (m, 2H), 6.78 (dd, *J* = 9.1, 2.2 Hz, 1H), 6.71 (d, *J* = 2.1 Hz, 1H), 3.82 (t, *J* = 6.3 Hz, 2H), 2.78 (t, *J* = 6.3 Hz, 2H). ^13^C NMR (75 MHz, CDCl_3_) δ 168.3, 144.4, 143.0, 134.2, 132.2, 128.7, 128.2, 127.7, 126.2, 123.3, 118.8, 115.6, 40.6, 39.9. HPLC–MS (gradient II) (M + H)^+^ = 354, *R*_t_ = 3.63 min (99%).

### General Procedure B for the Synthesis of **7**–**19**

The corresponding precursor with the amide group (1 equiv) was dissolved in CH_3_CN and the corresponding amine (1.1 equiv) dissolved in CH_3_CN was added dropwise. The base (Et_3_N or DIPEA, indicated in each case) (2 equiv) was added and the reaction was heated at 80 °C for 24 h. At the end of the reaction time the solvent was evaporated, the product was dissolved in EtOAc and the organic phase was washed with H_2_O and saturated NaCl solution and then dried over anhydrous Mg_2_SO_4_. The desiccant was filtered off, the volatiles were evaporated to dryness under vacuum and the resulting residue was purified by flash column chromatography using mixtures of solvents as eluents as indicated in each case.

#### *N*-(2-((5-Chloro-2-nitrophenyl)­thio)­phenyl)-2-(pyrrolidin-1-yl)­acetamide (**7**)

The title compound was prepared by reaction of 2-chloro-*N*-(2-((5-chloro-2-nitrophenyl)­thio)­phenyl)­acetamide (**3**) (0.2 mmol, 71 mg), pyrrolidine (0.22 mmol, 18 μL), Et_3_N (0.4 mmol, 56 μL) and CH_3_CN as solvent (2 mL) according to procedure B. Purification: CH_2_Cl_2_/MeOH (9:1). Yield: 42 mg (53%) as a yellow solid. Mp 135–137 °C. ^1^H NMR (300 MHz, DMSO-*d*_6_) δ 10.06 (s, 1H), 8.49 (d, *J* = 8.3 Hz, 1H), 8.39 (d, *J* = 8.9 Hz, 1H), 7.82–7.62 (m, 2H), 7.54 (dd, *J* = 8.9, 2.2 Hz, 1H), 7.33 (td, *J* = 7.6, 1.2 Hz, 1H), 3.16 (s, 2H), 2.30 (d, *J* = 5.5 Hz, 4H), 1.61–1.35 (m, 4H). ^13^C NMR (75 MHz, DMSO-*d*_6_) δ 169.3, 143.4, 140.7, 140.5, 138.6, 137.9, 133.3, 128.8, 126.8, 125.9, 125.6, 120.9, 117.2, 59.2, 53.9 (2C), 23.8 (2C). HRMS (ESI) *m*/*z*: calc. for C_18_H_19_ClN_3_O_3_S [M + H]^+^ 392.0830; found 392.0826. HPLC-MS (gradient II) (M + H)^+^ = 392, *R*_t_ = 2.71 min (99%).

#### *N*-(2-((5-Chloro-2-nitrophenyl)­thio)­phenyl)-2-(diethylamino)­acetamide (**8**)

The title compound was prepared by reaction of 2-chloro-*N*-(2-((5-chloro-2-nitrophenyl)­thio)­phenyl)­acetamide (**3**) (0.2 mmol, 71 mg), diethylamine (0.22 mmol, 23 μL), Et_3_N (0.4 mmol, 56 μL) and CH_3_CN as solvent (2 mL) according to procedure B. Purification: CH_2_Cl_2_/MeOH (9:1). Yield: 42 mg (53%) as a yellow solid. Mp 92–94 °C. ^1^H NMR (300 MHz, DMSO-*d*_6_) δ 10.21 (s, 1H), 8.56 (dd, *J* = 8.3, 1.2 Hz, 1H), 8.38 (d, *J* = 8.9 Hz, 1H), 7.77 (dd, *J* = 7.7, 1.5 Hz, 1H), 7.72–7.63 (m, 1H), 7.54 (dd, *J* = 8.9, 2.2 Hz, 1H), 7.32 (td, *J* = 7.6, 1.3 Hz, 1H), 6.52 (d, *J* = 2.2 Hz, 1H), 3.02 (s, 2H), 2.29 (q, *J* = 7.1 Hz, 4H), 0.69 (t, *J* = 7.1 Hz, 6H). ^13^C NMR (75 MHz, DMSO-*d*_6_) δ 170.5, 143.9, 140.7, 140.6, 138.5, 138.3, 133.5, 128.9, 127.0, 126.1, 125.5, 120.5, 116.8, 57.9, 48.5 (2C), 12.1 (2C). HRMS (ESI) *m*/*z*: calc. for C_18_H_21_ClN_3_O_3_S [M + H]^+^ 394.0987; found 394.0984. HPLC-MS (gradient II) (M + H)^+^ = 394, *R*_t_ = 2.76 min (99%).

#### *N*-(2-((5-Chloro-2-nitrophenyl)­thio)­phenyl)-2-(piperidin-1-yl)­acetamide (**9**)

The title compound was prepared by reaction of 2-chloro-*N*-(2-((5-chloro-2-nitrophenyl)­thio)­phenyl)­acetamide (**3**) (0.2 mmol, 71 mg), piperidine (0.22 mmol, 22 μL), Et_3_N (0.4 mmol, 56 μL) and CH_3_CN as solvent (2 mL) according to procedure B. Purification: CH_2_Cl_2_/MeOH (9:1). Yield: 32 mg (39%) as a yellow solid. Mp 123–125 °C. ^1^H NMR (300 MHz, DMSO-*d*_6_) δ 10.15 (s, 1H), 8.52 (dd, *J* = 8.3, 1.0 Hz, 1H), 8.40 (d, *J* = 8.9 Hz, 1H), 7.81–7.62 (m, 2H), 7.62–7.46 (m, 1H), 7.33 (td, *J* = 7.6, 1.3 Hz, 1H), 6.48 (d, *J* = 2.2 Hz, 1H), 2.99 (s, 2H), 2.25 (s, 4H), 1.20 (s, 6H). ^13^C NMR (75 MHz, DMSO-*d*_6_) δ 169.4, 143.6, 141.1, 140.7, 138.9, 138.2, 133.5, 128.9, 126.9, 125.8 (2C), 121.1, 117.0, 62.6, 54.5 (2C), 25.9 (2C), 23.5. HRMS (ESI) *m*/*z*: calc. for C_19_H_21_ClN_3_O_3_S [M + H]^+^ 406.0987; found 406.0982. HPLC-MS (gradient II) (M + H)^+^ = 406, *R*_t_ = 2.77 min (99%).

#### 2-(Benzylamine)-*N*-(2-((5-chloro-2-nitrophenyl)­thio)­phenyl)­acetamide (**10**)

The title compound was prepared by reaction of 2-chloro-*N*-(2-((5-chloro-2-nitrophenyl)­thio)­phenyl)­acetamide (**3**) (0.14 mmol, 50 mg), benzylamine (0.17 mmol, 20 μL), Et_3_N (0.4 mmol, 38.5 μL) and CH_3_CN as solvent (2 mL) according to procedure B. Purification: CH_2_Cl_2_/MeOH (9:1). Yield: 28 mg (47%) as a yellow solid. Mp 110–112 °C. ^1^H NMR (300 MHz, DMSO-*d*_6_) δ 10.32 (s, 1H), 8.50 (dd, *J* = 8.3, 1.3 Hz, 1H), 8.27 (d, *J* = 8.8 Hz, 1H), 7.73 (dd, *J* = 7.7, 1.5 Hz, 1H), 7.67 (ddd, *J* = 8.3, 7.4, 1.6 Hz, 1H), 7.43 (dd, *J* = 8.8, 2.2 Hz, 1H), 7.32 (td, *J* = 7.6, 1.4 Hz, 1H), 7.28–7.13 (m, 3H), 7.09–6.98 (m, 2H), 6.52 (d, *J* = 2.1 Hz, 1H), 3.19 (s, 2H), 2.45 (s, 2H). ^13^C NMR (75 MHz, DMSO-*d*_6_) δ 171.0, 143.9, 141.0, 140.4, 139.9, 138.8, 138.0, 133.3, 128.7 (2C), 128.7 (2C), 128.7, 126.8, 126.4, 126.3, 125.7, 121.1, 117.8, 52.8, 51.3. HRMS (ESI) *m*/*z*: calc. for C_21_H_19_ClN_3_O_3_S [M + H]^+^ 428.0830; found 428.0835. HPLC-MS (gradient II) (M + H)^+^ = 428, *R*_t_ = 3.03 min (99%).

#### 2-((2-(1-Benzylpiperidin-4-yl)­ethyl)­amino)-*N*-(2-((5-chloro-2-nitrophenyl)­thio)­phenyl)­acetamide (**11**)

The title compound was prepared by reaction of 2-chloro-*N*-(2-((5-chloro-2-nitrophenyl)­thio)­phenyl)­acetamide (**3**) (0.2 mmol, 71 mg), 2-(1-benzylpiperidin-4-yl)­ethan-1-amine (0.22 mmol, 48 mg), Et_3_N (0.4 mmol, 56 μL) and CH_3_CN as solvent (2 mL) according to procedure B. Purification: CH_2_Cl_2_/MeOH (9:1). Yield: 64 mg (60%) as a yellow solid. Mp 118–120 °C. ^1^H NMR (300 MHz, DMSO-*d*_6_) δ 8.53 (dd, *J* = 8.3, 1.3 Hz, 1H), 8.35 (d, *J* = 8.8 Hz, 1H), 7.76–7.59 (m, 2H), 7.49 (dd, *J* = 8.8, 2.2 Hz, 1H), 7.40–7.17 (m, 6H), 6.50 (d, *J* = 2.2 Hz, 1H), 3.40 (s, 2H), 3.12 (s, 2H), 2.79–2.65 (m, 2H), 2.21 (t, *J* = 7.1 Hz, 2H), 1.90–1.74 (m, 2H), 1.42 (d, *J* = 11.2 Hz, 2H), 1.13–0.83 (m, 5H). ^13^C NMR (75 MHz, DMSO-*d*_6_) δ 171.1, 143.8, 141.1, 140.4, 139.2, 138.9, 138.0, 133.3, 129.1 (2C), 128.8, 128.5 (2C), 127.2, 126.9, 126.2, 125.6, 120.9, 117.5, 62.9, 53.7 (2C), 53.2, 47.5, 36.6, 33.5, 32.3 (2C). HRMS (ESI) calc. for C_28_H_32_ClN_4_O_3_S [M + H]^+^ 539.1878; found 539.1873. HPLC-MS (gradient II) (M + H)^+^ = 539, *R*_t_ = 2.40 min (95%).

#### *N*-(2-((5-Chloro-2-nitrophenyl)­thio)­phenyl)-3-(pyrrolidin-1-yl)­propanamide (**12**)

The title compound was prepared by reaction of 3-chloro-*N*-(2-((5-chloro-2-nitrophenyl)­thio)­phenyl)­propanamide (**4**) (0.14 mmol, 50 mg), pyrrolidine (0.36 mmol, 14.1 μL), Et_3_N (0.28 mmol, 39 μL) and CH_3_CN as solvent (2 mL) according to procedure B. Purification: CH_2_Cl_2_/MeOH (99:1). Yield: 70 mg (51%) as a yellow solid. Mp 100–102 °C. ^1^H NMR (300 MHz, DMSO-*d*_6_) δ 10.25 (s, 1H), 8.29 (d, *J* = 8.8 Hz, 1H), 7.93 (d, *J* = 8.1 Hz, 1H), 7.70–7.55 (m, 2H), 7.48 (dt, *J* = 8.8, 3.3 Hz, 1H), 7.34 (td, *J* = 7.6, 1.3 Hz, 1H), 6.56 (dd, *J* = 6.3, 2.2 Hz, 1H), 3.02–2.64 (m, 6H), 2.56 (dd, *J* = 11.7, 5.0 Hz, 2H), 1.80–1.60 (m, 4H). ^13^C NMR (75 MHz, DMSO-*d*_6_) δ 170.2, 143.9, 141.5, 140.0, 139.9, 137.6, 132.3, 128.4, 127.0, 126.9, 126.5, 126.1, 122.1, 53.5 (2C), 50.9, 33.8, 23.3 (2C). HRMS (ESI) *m*/*z*: calc. for C_19_H_21_ClN_3_O_3_S [M + H]^+^ 406.0987; found 406.0982. HPLC-MS (gradient II) (M + H)^+^ = 406, *R*_t_ = 2.67 min (99%).

#### *N*-(2-((5-Chloro-2-nitrophenyl)­thio)­phenyl)-3-(diethylamino)­propanamide (**13**)

The title compound was prepared by reaction of 3-chloro-*N*-(2-((5-chloro-2-nitrophenyl)­thio)­phenyl)­propanamide (**4**) (1.35 mmol, 500 mg), diethylamine (1.48 mmol, 195 μL), Et_3_N (2.69 mmol, 469 μL) and CH_3_CN as solvent (8 mL) according to procedure B. Purification: CH_2_Cl_2_/MeOH (99:1). Yield: 309 mg (56%) as an orange oil. ^1^H NMR (300 MHz, CDCl_3_) δ 11.19 (s, 1H), 8.60 (dd, *J* = 8.4, 1.3 Hz, 1H), 8.29 (d, *J* = 8.8 Hz, 1H), 7.67–7.56 (m, 2H), 7.31–7.22 (m, 2H), 6.69 (d, *J* = 2.1 Hz, 1H), 2.77–2.69 (m, 2H), 2.58–2.48 (m, 6H), 0.93 (t, *J* = 7.2 Hz, 6H). ^13^C NMR (75 MHz, CDCl_3_) δ 171.9, 143.2, 142.4, 141.4, 140.8, 137.6, 132.5, 127.4, 126.9, 125.6, 125.4, 123.4, 118.0, 47.6, 45.8 (2C), 34.0, 10.2 (2C). HRMS (ESI) *m*/*z*: calc. for C_19_H_23_ClN_3_O_3_S [M + H]^+^ 408.1143; found 408.1144. HPLC-MS (gradient I) (M + H)^+^ = 408, *R*_t_ = 4.19 min (97%).

#### *N*-(2-((5-Chloro-2-nitrophenyl)­thio)­phenyl)-3-(phenethylamino)­propanamide (**14**)

The title compound was prepared by reaction of 3-chloro-*N*-(2-((5-chloro-2-nitrophenyl)­thio)­phenyl)­propanamide (**4**) (0.3 mmol, 107 mg), 2-phenethylamine (0.36 mmol, 45.3 μL), Et_3_N (0.6 mmol, 84 μL) and CH_3_CN as solvent (3 mL) according to procedure B. Purification: CH_2_Cl_2_/MeOH (99:1). Yield: 70 mg (51%) as a yellow solid. Mp 85–87 °C. ^1^H NMR (300 MHz, DMSO-*d*_6_) δ 10.60 (s, 1H), 8.07–7.95 (m, 2H), 7.62 (dt, *J* = 5.1, 2.5 Hz, 1H), 7.60–7.51 (m, 1H), 7.34–7.22 (m, 4H), 7.21–7.13 (m, 3H), 7.07–7.01 (m, 1H), 2.83–2.62 (m, 6H), 2.42 (t, *J* = 6.3 Hz, 2H). ^13^C NMR (75 MHz, DMSO-*d*_6_) δ 171.4, 146.5, 144.7, 141.4, 140.1, 137.1, 131.9, 128.9 (2C), 128.7 (2C), 128.1, 127.3, 126.9, 126.4, 126.3, 125.5, 125.1, 121.0, 50.5, 44.9, 35.8, 35.5. HRMS (ESI) *m*/*z*: calc. for C_23_H_23_ClN_3_O_3_S [M + H]^+^ 456.1143; found 456.1147. HPLC-MS (gradient II) (M + H)^+^ = 456, *R*_t_ = 3.12 min (95%).

#### 3-((2-(1-Benzylpiperidin-4-yl)­ethyl)­amino)*-N-*(2-((5-chloro-2-nitrophenyl)­thio)­phenyl)­propanamide (**15**)

The title compound was prepared by reaction of 3-chloro-*N*-(2-((5-chloro-2-nitrophenyl)­thio)­phenyl)­propanamide (**4**) (0.71 mmol, 263 mg), 2-(1-benzylpiperidin-4-yl)­ethan-1-amine (0.78 mmol, 170 mg), Et_3_N (1.42 mmol, 198 μL) and CH_3_CN as solvent (4 mL) according to procedure B. Purification: CH_2_Cl_2_/MeOH (9:1). Yield: 254 mg (65%) as an orange oil. ^1^H NMR (300 MHz, CDCl_3_) δ 10.88 (s, 1H), 8.50 (dd, *J* = 8.7, 1.3 Hz, 1H), 8.21 (d, *J* = 8.8 Hz, 1H), 7.62–7.55 (m, 2H), 7.34 (m, 4H), 7.26–7.19 (m, 3H), 6.69 (d, *J* = 2.1 Hz, 1H), 3.51 (s, 2H), 2.94–2.83 (m, 2H), 2.82–2.75 (m, 2H), 2.53–2.44 (m, 2H), 2.43–2.35 (m, 2H), 2.00–1.89 (m, 2H), 1.62–1.52 (m, 2H), 1.31–1.18 (m, 5H). ^13^C RMN (75 MHz, CDCl_3_) δ 171.7, 143.5, 142.0, 141.3, 140.1, 137.4, 132.6, 129.4 (2C), 128.3, 127.3, 127.2 (3C), 125.7, 125.3, 123.3, 118.2, 63.5, 53.8 (2C), 46.6, 44.9, 36.2, 33.8, 32.3 (2C). HRMS (ESI) *m*/*z*: calc. for C_29_H_34_ClN_4_O_3_S [M + H]^+^ 553.2035; found 553.2067. HPLC-MS (gradient I) (M + H)^+^ = 553, *R*_t_ = 3.23 min (99%).

#### 2-((2-(1-Benzylpiperidin-4-yl)­ethyl)­amino)-*N*-(2-((5-chloro-2-nitrophenyl)­amino)­phenyl)­acetamide (**16**)

The title compound was prepared by reaction of 2-chloro-*N*-(2-((5-chloro-2-nitrophenyl)­amino)­phenyl)­acetamide (**5**) (0.68 mmol, 230 mg), 2-(1-benzylpiperidin-4-yl)­ethan-1-amine (0.74 mmol, 162 mg), Et_3_N (1.36 mmol, 189 μL) and CH_3_CN as solvent (5 mL) according to procedure B. Purification: CH_2_Cl_2_/MeOH (9:1). Yield: 243 mg (68%) as a red oil. ^1^H NMR (300 MHz, CDCl_3_) δ 9.74 (s, 1H), 9.22 (s, 1H), 8.40 (dd, *J* = 8.2, 1.4 Hz, 1H), 8.17 (d, *J* = 9.1 Hz, 1H), 7.49–7.29 (m, 7H), 7.21 (td, *J* = 7.6, 1.4 Hz, 1H), 6.75 (dd, *J* = 9.1, 2.2 Hz, 1H), 6.67 (d, *J* = 2.2 Hz, 1H), 3.65 (s, 2H), 3.31 (s, 2H), 3.08–2.89 (m, 2H), 2.53–2.39 (m, 2H), 2.16–1.97 (m, 2H), 1.63–1.51 (m, 2H), 1.44–1.28 (m, 2H), 1.20–1.11 (m, 3H). ^13^C NMR (75 MHz, CDCl_3_) δ 170.3, 144.6, 143.1, 134.7, 131.7, 130.0, 129.0 (2C), 128.6 (2C), 128.2, 127.9, 127.0, 125.2, 121.8, 118.5, 115.4, 62.7, 53.2, 47.9, 36.5, 33.2 (2C), 31.2 (2C), 29.8. HRMS (ESI) *m*/*z*: calc. for C_28_H_33_ClN_5_O_3_ [M + H]^+^ 522.2266; found 522.2254. HPLC-MS (gradient II) (M + H)^+^ = 522, *R*_t_ = 2.38 min (99%).

#### *N*-(2-((5-Chloro-2-nitrophenyl)­amino)­phenyl)-3-(pyrrolidin-1-yl)­propanamide (**17**)

The title compound was prepared by reaction of 3-chloro-*N*-(2-((5-chloro-2-nitrophenyl)­amino)­phenyl)­propanamide (**6**) (1.41 mmol, 500 mg), pyrrolidine (1.55 mmol, 129 μL), Et_3_N (2.82 mmol, 392 μL) and CH_3_CN as solvent (10 mL) according to procedure B. Purification: CH_2_Cl_2_/MeOH (95:5). Yield: 165 mg (30%) as an orange solid. Mp 173–175 °C. ^1^H NMR (300 MHz, CDCl_3_) δ 10.90 (s, 1H), 9.28 (s, 1H), 8.16 (d, *J* = 9.1 Hz, 1H), 8.10 (dd, *J* = 8.2, 1.4 Hz, 1H), 7.38–7.30 (m, 1H), 7.25–7.16 (m, 2H), 6.81 (d, *J* = 2.2 Hz, 1H), 6.74 (dd, *J* = 9.1, 2.2 Hz, 1H), 2.72 (dd, *J* = 6.6, 4.9 Hz, 2H), 2.57 (dd, *J* = 6.5, 4.7 Hz, 2H), 2.48–2.38 (m, 4H), 1.59–1.50 (m, 4H). ^13^C NMR (75 MHz, CDCl_3_) δ 171.6, 144.6, 142.8, 135.1, 131.9, 129.3, 128.1, 128.0, 127.0, 125.5, 124.3, 118.3, 115.9, 53.4 (2C), 51.8, 34.8, 23.6 (2C). HRMS (ESI) *m*/*z*: calc. for C_19_H_22_ClN_4_O_3_ [M + H]^+^ 389.1375; found 389.1380. HPLC-MS (gradient II) (M + H)^+^ = 389, *R*_t_ = 2.63 min (99%).

#### *N*-(2-((5-Chloro-2-nitrophenyl)­amino)­phenyl)-3-(diethylamino)­propanamide (**18**)

The title compound was prepared by reaction of 3-chloro-*N*-(2-((5-chloro-2-nitrophenyl)­amino)­phenyl)­propanamide (**6**) (3.82 mmol, 1.35 g), diethylamine (4.2 mmol, 435 μL), DIPEA (7.64 mmol, 1.33 mL) and CH_3_CN as solvent (15 mL) according to procedure B. Purification: CH_2_Cl_2_/MeOH (9:1). Yield: 951 mg (64%) as an orange solid. Mp 109–111 °C. ^1^H NMR (300 MHz, CDCl_3_) δ 10.70 (s, 1H), 9.30 (s, 1H), 8.12 (d, *J* = 9.1 Hz, 1H), 7.97 (dd, *J* = 8.0, 1.5 Hz, 1H), 7.35–7.25 (m, 2H), 7.24–7.16 (m, 1H), 6.79 (d, *J* = 2.2 Hz, 1H), 6.71 (dd, *J* = 9.1, 2.2 Hz, 1H), 2.83 (t, *J* = 6.0 Hz, 2H), 2.64 (t, *J* = 6.1 Hz, 2H), 2.58 (q, *J* = 7.2 Hz, 4H), 0.96 (t, *J* = 7.2 Hz, 6H). ^13^C NMR (75 MHz, CDCl_3_) δ 171.2, 144.5, 142.6, 134.6, 131.9, 129.8, 128.0, 127.9, 127.2, 125.7, 124.5, 118.1, 115.8, 48.0, 45.9 (2C), 32.9, 10.0 (2C). HRMS (ESI) *m*/*z*: calc. for C_19_H_24_ClN_4_O_3_ [M + H]^+^ 391.1531; found 391.1542. HPLC-MS (gradient I) (M + H)^+^ = 391, *R*_t_ = 4.07 min (98%).

#### *N*-(2-((5-Chloro-2-nitrophenyl)­amino)­phenyl)-3-(phenethylamino)­propanamide (**19**)

The title compound was prepared by reaction of 3-chloro-*N*-(2-((5-chloro-2-nitrophenyl)­amino)­phenyl)­propanamide (**6**) (1.24 mmol, 440 mg), 2-phenethylamine (1.37 mmol, 171 μL), Et_3_N (2.48 mmol, 345 μL) and CH_3_CN as solvent (10 mL) according to procedure B. Purification: CH_2_Cl_2_/MeOH (9:1). Yield: 36 mg (7%) as a red solid. Mp 156–158 °C. ^1^H NMR (300 MHz, CDCl_3_) δ 10.19 (s, 1H), 9.43 (s, 1H), 9.03 (s, 1H), 8.16 (d, *J* = 9.1 Hz, 1H), 7.66–7.54 (m, 1H), 7.51–7.43 (m, 1H), 7.37–7.21 (m, 7H), 6.86 (dd, *J* = 9.1, 2.2 Hz, 1H), 6.70 (d, *J* = 2.2 Hz, 1H), 3.26–3.09 (m, 4H), 3.02–2.90 (m, 2H), 2.79 (t, *J* = 7.2 Hz, 2H). ^13^C NMR (75 MHz, CDCl_3_) δ 168.8, 143.5, 140.7, 137.2, 133.0, 131.8, 131.6, 128.6 (4C), 128.3, 127.1, 126.9, 126.8, 126.4, 125.7, 117.3, 114.7, 47.8, 42.7, 31.5 (2C). HRMS (ESI) *m*/*z*: calc. For C_23_H_24_ClN_4_O_3_ [M + H]^+^ 439.1531; found 439.1536. HPLC-MS (gradient II) (M + H)^+^ = 439, *R*_t_ = 2.42 min (95%).

### Antiviral Activity in EBOV-GP Pseudotyped Viruses

#### Cell Lines

Human embryonic kidney cells (293*T*/17; ATCC-CRL-11268), baby hamster kidney cells (BHK-21/WI-2, Kerafast # EH1011) and African Green Monkey Cell Line (VeroE6) were cultured in Dulbecco’s modified Eagle medium (DMEM) supplemented with 10% heat-inactivated fetal bovine serum (FBS), 25 μg/mL gentamycin and 2 mM l-glutamine.

#### Construction of Ebola-GP-Y517S Mutants

Generation of plasmid with single-point mutation Y517S in Ebola virus glycoprotein: Plasmid encoding the EBOV-GP Y517S was carried out by following the Q5 Site-Directed Mutagenesis standard protocol (New England BioLabs).

Primer pairs containing the mutation of interest were designed using New England BioLabs web-based design program (listed below)

EBO GP Y517S_F CAATTTACATTCCTGGACTACTCAGG

EBO GP Y517S _R GGGTTGCATTTGGGTTGA

Mutant construction was confirmed by sequencing, using an ABI PRISM 3100 Genetic Analyzer (Applied Biosystems) and posterior sequence analysis by Geneious R6 bioinformatics software. All the plasmids were prepared with HiPure Plasmid Filter Maxiprep (Invitrogen) and quantified by spectrophotometry (NanoDrop).

#### Production of Recombinant Viruses with EBOV-GP or VSV-G with the VSV Backbone

VSV-G pseudotyped replication deficient rVSV-luc recombinant viruses were produced to test the inhibitory activity of selected compounds. The viral construction was pseudotyped with EBOV-GP (strain Mayinga, GeneBank: U23187.1) or vesicular stomatitis virus envelope GP (VSV-G) and expressed luciferase as a reporter of the infection. BHK-21 were transfected to express EBOV-GP using Lipofectamine 3000 (Thermo Fisher Scientific, Madrid, Spain) and after 24 h cells were inoculated with a replication-deficient rVSV-luc (MOI: 3–5) that contains firefly luciferase instead of the VSV-G open reading frame, rVSVΔG-luciferase (G*ΔG-luciferase; Kerafast). After 1 h incubation at 37 °C, the inoculum was removed, cells were washed intensively with PBS and then the medium was added. Pseudotyped particles were harvested 20 h postinoculation, clarified from cellular debris by centrifugation and stored at −80 °C. Infectious titers were estimated as tissue culture infectious dose per mL by limiting dilution of the Ebola-GP rVSV-luc-containing supernatants on Vero E6 cells. Luciferase activity was determined by luciferase assay (Steady-Glo Luciferase Assay System, Promega) in a GloMax Navigator Microplate Luminometer (Promega).

#### Screening of Selected Compounds

All the compounds tested in this work were initially resuspended in DMSO at 1 mM.

Screening of selected compounds as EBOV-GP pseudotyped virus entry inhibitors was performed using VeroE6 cells (2 × 10^4^ cells/well) in 96-well plates.

VeroE6 cells were incubated at 37 °C for 1 h with the compounds and then challenged with 5000 TCID (Tissue Culture Infective Dose) of recombinant viruses. After 24 h of incubation, cells were washed with PBS, lysed by addition of Steady-Glo Lysis Buffer (Promega) and light measured in a GloMax Navigator Microplate Luminometer (Promega).

Compounds that inhibited virus infection by more than 75% at a final concentration of 10 μM were further analyzed for potency, selectivity and cytotoxicity. For these compounds, the range of concentrations tested was 10 nM–10 μM. As a control for selectivity, infection with VSV-G pseudoviruses was performed in the same conditions (Table S1 of Supporting Information).

#### Toxicity Analysis of Compounds

VeroE6 (2 × 10^4^) cells were seeded in a 96-well plate and incubated with DMEM containing each compound at concentrations ranging from 0 to 200 μM. After 24 h, cell viability was measured by CellTiter-Glo Luminescent Cell Viability Assay (Promega).

Cell viability was reported as the percentage of luminescence in treated cells relative to nontreated cells.

CC_50_ was calculated and nontoxic working concentrations (over 80% cell viability) used to test the activities of these compounds on EBOV-GP-pseudotyped infection.

#### Statistical Analysis

The values of EC_50_ inhibition of the infection presented on the table correspond to the mean of 3 independent experiments. The EC_50_s values were estimated using GraphPad Prism v6.0 with a 95% confidence interval and settings for normalize dose–response curves.

#### Effect of the Ebola GP Y517S Mutation on the Inhibitory Capacity of Selected Compounds

293T cells were infected with EBOV-GP pseudotypes or with the mutant Y517S in the presence of selected compounds (5 μM) previously incubated at 37 °C for 1 h with these cells. 48 h later, cells were lysed and light measured.

As control compounds imipramine at 10 μM and toremifene at 1 μM were used for Y517S mutant.

### Antiviral Efficacy during EBOV Infection

A total of 1 × 10^5^ Vero E6 cells were seeded in 24-well plate and subsequently infected with the EBOV Mayinga variant at a multiplicity of infection (MOI) of 0.1. After a one-hour incubation, unbound virus was removed by discarding the supernatant, followed by a single wash with 500 μL of PBS. Meanwhile, the compounds, initially dissolved in DMSO at a concentration of 10 mM, were prepared and incorporated into DMEM containing 5% FBS and methylcellulose. The compounds were tested at concentrations ranging from 0.1 μM to 50 μM. After their addition, the cells were incubated at 37 °C with 5% CO_2_ for 3 days. The concentration of infectious virus particles in the cell culture supernatant was then determined using an immunofocus assay. Briefly, the supernatant was removed, and cells were fixed with 4% paraformaldehyde for 1 h. After fixation, the plates were thoroughly washed between each step. Cells were then permeabilized for 30 min with 0.5% Triton X-100 in PBS, and blocked with 5% FBS in PBS for 1 h. A polyclonal mouse anti-EBOV antibody (1:5000 in 2.5% blocking solution), was then added and incubated overnight. The secondary antibody, a peroxidase-conjugated sheep antimouse IgG (H+L) was then applied for 1 h at 1:5000 in 5% blocking solution. Foci were detected by adding tetramethylbenzidine (1:3 in distilled water) for 30 min or until spots developed, after which the reagent was discarded and foci were counted.

The concentrations required to reduce viral titer by 50% (EC_50_) were determined from dose–response curves using GraphPad Prism 9 with a 95% confidence interval.

### Liver Microsome Stability Assay

Mouse or Human liver microsomes and reduced nicotinamide adenine dinucleotide phosphate (NADPH), warfarine and MgCl_2_ were purchased from Fisher Scientific SL. Verapamil used as control was purchased from Sigma-Aldrich. Microsome stability was tested by incubating 10 μM of test compounds and verapamil (as system control) with 1.0 mg/mL hepatic microsomes (pooled human liver microsomes and pooled mouse (CD-1) liver microsomes) in 0.1 M potassium phosphate buffer (pH 7.4) with MgCl_2_ 5 mM. The reaction was initiated by adding NADPH (1 mM final concentration). Aliquots of 150 μL were collected at defined time points (0, 5, 15, 30, 45, and 60 min) and added to cold acetonitrile (150 μL) containing an internal standard (5 μg/mL warfarin) to stop the reaction and precipitate the protein. The samples were centrifuged at 4 °C for 15 min and the loss of parent compounds was analyzed by high performance liquid chromatography coupled to mass spectrometry (HPLC-MS) using single ion mode (SIM) detection. The instrument used was a HPLC Surveyor with PDA Surveyor plus UV–vis detector coupled to a Thermo Mod. Finnigan LXQ with positive electrospray ionization (ESI). The column used was a ZORBAX SC-C18 (3.5 μm, 4.6 mm × 50 mm). Gradient elution used was H_2_O/CH_3_CN containing 0.1% of formic acid from 100/0 to 0/100 for 7 min with an 800 μL/min flow. Data were log transformed and represented as half-life. All experiments were conducted in duplicates.

### STD–NMR Experiments

All NMR spectra were acquired at 298 K using a Bruker AVANCE 600 MHz spectrometer and processed with TOPSIN 4.1.4 software (Bruker, SA). NMR samples were prepared in deuterated Tris buffer 50 mM, NaCl 50 mM, pH 7.4. EBOV-GP was produced according to a previous described procedure.^22^ STD experiments were performed with a 100:1 ligand-protein molar ratio and saturation time of 2 s. The protein was saturated in the aliphatic region of the spectrum at 0.8 ppm (on-resonance experiment), and the off-resonance experiment was saturated at 100 ppm. Relative STD effects were calculated by comparing the intensity of the signals in the STD–NMR spectrum (ISTD) with signal intensities of the reference spectrum (off-resonance). The STD signal with high intensity was set to 100%, and other STD signals were calculated accordingly. Control STD–NMR experiments were performed using an identical experimental setup and the same ligand concentration but in the absence of the protein. Competition STD experiments were acquired with the same conditions as above. Increasing concentrations of imipramine were added in 1:1, 1:2, 1:3 and 1:4 ratios.

### Molecular Dynamics (MD) Simulations

MD simulations were conducted to investigate the structure and behavior of the protein bound to compound **16**. To achieve this, the GP system was constructed using the protein bound to toremifene, as documented in PDB entry 5JQ7.^16^ The sulfide derivative was docked onto the optimized X-ray structure, with the binding site grid set at the toremifene position.^36^ The protein’s N- and C-termini were neutralized with acetyl (ACE) and *N*-methyl (NME) caps, and 15 disulfide bonds (C53_A‑C_-C609_A‑C_, C108_A‑C_-C135_A‑C_, C121_A‑C_- C147_A‑C_, C511_A‑C_-C556_A‑C_, C601_A‑C_-C609_A‑C_) were defined to create the functional homotrimer.

The system was immersed in an octahedral box of TIP3P water molecules,^37^ with ionizable residues in their standard protonation state at physiological pH. The model protein, including ∼21,000 water molecules, totaled around 80,000 atoms. Simulations used the NPT ensemble for equilibration and the NVT ensemble for production runs, with periodic boundary conditions and Ewald sums for long-range electrostatic interactions. The Amber ff14SB force field^38^ was used for protein and the GAFF force field^39^ in conjugation with restrained electrostatic potential-fitted (RESP)^40^ partial atomic charges derived from B3LYP/6-31G­(d) calculations was used for ligand. All the simulations were executed using Amber20.^41^

The initial system was minimized in multiple steps: Initially, the positions of all hydrogen atoms in the protein underwent refinement via energy minimization (2000 cycles of steepest descent +8000 cycles of conjugate gradient). Subsequently, this approach was extended to minimize the positions of water molecules and counterions. Finally, all atoms within the system underwent energy minimization (4000 cycles for steepest descent +1000 cycles of conjugate gradient). The equilibration process encompassed six steps. The system was initially heated from 0 to 100 K in 20 ps (NVT ensemble), followed by four thermalization steps to elevate the temperature from 100 to 300 K (50 ps/step, NPT conditions). A concluding 5 ns step was conducted to equilibrate the system’s density at a constant temperature (300 K) and pressure (1 atm). Distance constraints were used during equilibration to stabilize the ligand’s position preventing any artifactual movements in the initial stages and gradually removed in MD simulations.^42,43^ Three protein–ligand complex replicas were simulated for 500 ns.

For the analysis, the CPPTRAJ20 ^44^ module’s rmsd, and rmsf commands were utilized to assess the protein and ligand’s stability.

### Essential Dynamics (ED)

This method was used to pinpoint the key motions resulting from structural variations observed in MD simulations. In ED analysis, the movements along individual modes are analyzed and visualized independently, allowing for the identification of the primary collective motions during the simulations. This involves creating and diagonalizing a positional covariance matrix to derive the collective deformation modes, known as eigenvectors, with the corresponding eigenvalues indicating each motion’s contribution to the protein’s overall structural variance. In our research, ED analysis utilized 25,000 snapshots from the 500 ns of each simulation, focusing exclusively on the backbone atoms. These calculations were executed using the PCAsuite program, available at http://www.mmb.irbbarcelona.org/software/pcasuite/pcasuite.html, and integrated into the pyPCcazip tool suite.^45^

### Interaction Energy Network

Networks of local interactions are intrinsically connected to how proteins structurally respond to external factors.^46^ For our analysis, we employed the Weighted Implementation of Suboptimal Path (WISP)^31^ to investigate the allosteric network. This approach allowed us to conduct a dynamic network analysis to explore how the binding of a ligand in an allosteric cavity can influence another binding site. WISP specifically relies on the dynamic interdependence among protein residues. Each amino acid is considered a node, located at the residue’s center-of-mass, and the interdependence among nodes is represented by a connecting edge with a numerical value indicating its strength. This interdependence is derived from an *N* × *N* matrix *C* (where *N* is the number of nodes) containing values corresponding to the weights of each edge, reflecting the correlated motion between node pairs. The weight of the edge connecting nodes *i* and *j* is denoted as *W*_*ij*_ = −log (|*C*_*ij*_|), with highly correlated or anticorrelated motions indicated by low *W*_*ij*_ values. This analysis was conducted for the 500 ns of the MD simulations.

### Inhibitory Effect of Compounds on EBOV-GPcl/NPC1-Domain C Interaction

*Cleaved EBOV-GP (EBOV-GPcl)* was generated in vitro using the bacterial protease thermolysin (250 μg/mL) (Sigma-Aldrich, St.Louis, MO) for 1 h at 37 °C, and stopping the reaction by adding the metalloprotease inhibitor phosphoramidon (1 mM) (Sigma-Aldrich) for 20 min on ice.

#### NPC1-Domain C Construct (Plasmid)

A cassette vector based on Homo sapiens NPC1-mRNA NM-000271 encoding the following sequence elements were synthesized on a pcDNA3 plasmid: Signal peptide (residues 1–24); domain C (residues 373–620); the first transmembrane domain (residues 267–295); Gly-Gly-Gly-Ser linker, and a triple Flag tag GeneArt (Thermo Fisher).

#### Expression, Purification, and Detection of NPC1-Domain C-Flag Fusion Protein

HEK293T cells (ATCC–CRL-11268) were transfected using Lipofectamine 3000 (Thermo Fisher) with the plasmid encoding NPC1-domain C-Flag. 36 h post transfection, cells were washed, lysed and collected (Cell Lytic M-C2978, Sigma-Aldrich).

Proteins from the cell lysate were purified by affinity chromatography using an anti-Flag-M2 agarose column according to the manufacturer’s instructions (Sigma-Aldrich).

Detection of NPC1-domain C-Flag protein was performed by Western blot using an Anti-Flag M2- Peroxydase (1:1000) monoclonal antibody (Sigma-Aldrich).

#### EbolaGP-NPC1 Domain C Binding ELISAs

NPC1-domain C concentrations used in the ELISAs were normalized using Micro BCA protein assay kit (Thermo).

Thermolysin-cleaved HIV-EBOV GP particles were captured onto high-binding 96-well ELISA plates (Corning, Corning, NY) using a conformation-specific anti-EBOV GP monoclonal antibody KZ52 (6.23 μg/mL).

Unbound viral particles were washed off, and purified Flag-tagged soluble NPC1-domain C (10 μg/mL) was added in the presence or not (control) of each compound (50 μM).

After that, bound flag-tagged proteins were detected with an anti-Flag antibody covalently conjugated to horseradish peroxidase (HRP) (1:5000) (Sigma-Aldrich). Finally, absorbance at 450 nm was measured after addition of TMB substrate.

### Prediction of the Blood–Brain Barrier (BBB) Penetration Using the Parallel Artificial Membrane Permeability Assay (PAMPA)^33^

Ten commercial drugs of known BBB permeability, namely caffeine, enoxacin, hydrocortisone, desipramine, ofloxacin, piroxicam, testosterone, promazine, verapamil and atenolol, acquired from Sigma-Aldrich, Merk and Fluka, were used as controls in each experiment to validate the analysis (Table S2 and Figure S3 from the Supporting Information). Controls and tested derivatives were dissolved in 5 mL of the experimental buffer (phosphate buffer saline solution at pH 7.4 (PBS)/EtOH (70:30 respectively)). The donor 96-well plate (Millipore, catalog no. MAIPS4510) was filled with 180 μL of each filtered compound solution after being coated with 5 μL of porcine brain lipid in dodecane (20 mg/mL, Sigma-Aldrich) (Avanti Polar Lipids, catalog no. 141101). The acceptor 96-well plate (Millipore, catalog no. 141101) was filled with 180 μL of the experimental buffer. Then, the donor plate was carefully put on the acceptor plate to form a “sandwich” for 2 h and 30 min at room temperature. During the incubation time, compounds diffused from the donor plate through the brain–lipid membrane into the acceptor plate. After incubation, the donor plate was removed, and the concentration of the compounds was determined in the acceptor and the donor plates by UV (Thermoscientific, Multiskan spectrum). Every sample was analyzed at 3–5 wavelengths in three wells and in two independent runs. Results are given as the mean ± standard deviation (SD) of the two runs.

## Supplementary Material







## Notes

Antiviral activity of selected derivatives against VSV-G-pseudotype virus (pVSV-G) (Table S1); permeability in the PAMPA-BBB assay (Table S2); representative binding mode of compound **17** within the cavity, formed between GP1 and GP2 (Figure S1); RMSF (Å) for the average of the three independent replicas of GP-**16** simulated system (Figure S2); linear correlation between experimental and reported permeability of ten commercial drugs used for the experiment validation in the PAMPA assay (Figure S3); HPLC chromatograms and NMR spectra of compounds **11–13** and **16–18** (Figures S4–S15) (PDF)
Molecular formula string (CSV)
Representative binding mode of compound **16** to GP1-GP2 (PDB)

## Abbreviations

ACE: acetyl
ACN: acetonitrile
BBB: blood–brain barrier
B3LYP: Becke, 3-parameter, Lee–Yang–Parr
CL_int_: intrinsic clearance
CC_50_: 50% cytotoxic concentration
*C*_max_: peak serum concentration
CNS: central nervous system
DMEM: Dulbecco’s modified Eagle medium
DMSO: dimethyl sulfoxide
EBOV: Orthoebolavirus zairense
EBOV-GP: Ebola virus glycoprotein
EC_50_: 50% efective concentration
ED: essential dynamics
ELISA: enzyme-linked immunosorbent assay
ESI: electrospray ionization
EVD: Ebola virus disease
FBS: fetal bovine serum
GAFF: general Amber force field
GP: glycoprotein
GPcl: cleaved glycoprotein
HIV: human immunodeficiency virus
HPLC–MS: high performance liquid chromatography coupled to mass spectrometry
HRMS-ESI: high resolution mass spectra
HRP: horseradish peroxidase
IC_50_: 50% inhibitory concentration
L: RNA-dependent RNA polymerase
MD: molecular dynamics
MOI: multiplicity of infection
MW: microwave irradiation
NADPH: nicotinamide adenine dinucleotide phosphate
NME: *N*-methyl
NP: nucleoprotein
NPC1: Niemann–Pick C1 receptor
NPC1-C: domain C of NPC1 (NPC1-C)
NVT: canonical ensemble with constant number of particles, volume, and temperature
PAMPA: parallel artificial membrane permeability assay
PDB ID: protein data bank identifier
Pe: permeability
pEBOV: EBOV-GP-pseudotyped virus
QTOF: quadrupole/time-of-flight
RBS: receptor binding site
RESP: restrained electrostatic potential
RMSD: root mean-square deviation
RMSF: root mean-square fluctuation
RNA: ribonucleic acid
rVSV: recombinant vesicular stomatitis virus
SAR: structure–activity relationships
SD: standard deviation
sGP: soluble glycoprotein
SI: selectivity index
SIM: single ion mode
STD–NMR: saturation transfer difference–nuclear magnetic resonance
TCID: tissue culture infective dose
TIP3P: transferable intermolecular potential with 3 point
*T*_max_: time to reach *C*_max_
*t*_1/2_: half-life time
VP 24: 30, 35, and 40, viral protein 24, 30, 35, and 40
VSV: vesicular stomatitis virus
VSV-G: vesicular stomatitis virus envelope GP
v/v: volume/volume
WISP: weighted implementation of suboptimal paths
wt: wild-type
