# Supplementary material for: From Diarylsulfides to Diarylamines: New Ebola Virus Entry Inhibitors with Improved Metabolic Stability
Source: J Med Chem. 2025 May 17;68(11):11786–800. doi: 10.1021/acs.jmedchem.5c00615 (PMC12169679; doi:10.1021/acs.jmedchem.5c00615)
Supplement: Supplementary file 1 [file jm5c00615_si_001.pdf]

SUPPORTING INFORMATION

FROM DIARYLSULFIDES TO DIARYLAMINES: NEW EBOLA VIRUS ENTRY INHIBITORS WITH IMPROVED METABOLIC STABILITY

Marcos Morales-Tenorio,[1,†] Fátima Lasala,[2, †] Alfonso Garcia-Rubia,[1] Elnaz Aledavood,[1] Michelle Heung,[3] Catherine Olal,[3] Beatriz Escudero-Pérez,[3] Paola Oquist,[4] Ángeles Canales,[4] Covadonga Alonso,[5] Ana Martínez,[1,6] César Muñoz-Fontela,[3] Rafael Delgado,[2,7,8,*] Carmen Gil[1,6,*]

[1]Centro de Investigaciones Biológicas Margarita Salas (CIB-CSIC), 28040 Madrid, Spain
[2]Instituto de Investigación Hospital 12 de Octubre, 28041 Madrid, Spain
[3]Bernhard Nocht Institute for Tropical Medicine, 20359 Hamburg, Germany
[4]Facultad de Ciencias Químicas, Universidad Complutense de Madrid, 28040 Madrid, Spain
[5]Dpt. Biotechnology, Instituto Nacional de Investigación y Tecnología Agraria y Alimentaria (INIA-CSIC), 28040 Madrid, Spain
[6]CIBERNED, Instituto Salud Carlos III, 28029 Madrid, Spain
[7]CIBERINFEC, Instituto Salud Carlos III, 28029 Madrid, Spain
[8]School of Medicine, Universidad Complutense de Madrid, 28040, Madrid, Spain

Table of content

- Page S2: Table S1: Antiviral activity of selected derivatives against VSV-G-pseudotype virus (pVSV-G) and Table S2: Permeability in the PAMPA-BBB assay.
- Page S3: Figure S1: Representative binding mode of compound **17** within the cavity, formed between GP1 and GP2.
- Page S4: Figure S2: RMSF (Å) for the average of the three independent replicas of GP-**16** simulated system and Figure S3: Linear correlation between experimental and reported permeability of ten commercial drugs used for the experiment validation in the PAMPA assay.
- Page S5-S10: HPLC chromatograms of compounds **11-13** and **16-18**.
- Page S11-S16: ^{1}H NMR and ^{13}C NMR spectra of compound **11-13** and **16-18**.

Table S1. Antiviral activity of selected derivatives against VSV-G-pseudotype virus (pVSV-G).

	%inh@10µM (pVSV-G)
**11**	38%@10µM
**12**	36%@10µM
**13**	-5%@10µM
**16**	-44%@10µM
**17**	-133%@10µM
**18**	9%@10µM

Table S2. Permeability (Pe 10^{-6} cm s^{-1}) in the PAMPA-BBB assay for 10 commercial drugs (used in the experiment validation) and the new derivatives tested with their predictive penetration into the central nervous system.

Compound	Literature[1]	***Pe* (10^{-6} cm s^{-1})[a]**	Prediction
Atenolol	0.8	0.3 ± 0.3	CNS−
Caffeine	1.3	1.5 ± 0.3	CNS−
Desipramine	12	13.9 ± 0.9	CNS+
Enoxacin	0.9	1.2 ± 0.2	CNS−
Hydrocortisone	1.9	2.6 ± 0.8	CNS−
Ofloxacin	0.8	1.4 ± 0.0	CNS−
Piroxicam	2.5	1.6 ± 0.7	CNS−
Promazine	8.8	9.1 ± 1.3	CNS+
Testosterone	17	19.0 ± 0.1	CNS+
Verapamil	16	15.0 ± 0.2	CNS+
**SC073**	-	11.4 ± 0.3	SNC +
**SC198**	-	11 ± 3	SNC +
**11**	-	6.2 ± 0.8	SNC +
**12**	-	16 ± 3	SNC +
**13**	-	14 ± 2	SNC +
**16**	-	9.7 ± 0.5	SNC +
**17**	-	15.9 ± 0.6	SNC +
**18**	-	18 ± 2	SNC +

[a]Data are presented as the mean ± SD of 2 independent experiments. CNS+ : able to cross the BBB by passive permeation. CNS− : not able to cross the BBB.

[1] Di, L.; Kerns, E. H.; Fan, K.; McConnell, O. J.; Carter, G. T. High throughput artificial membrane permeability assay for blood-brain barrier. *Eur. J. Med. Chem.* **2003,** *38*, 223–232.

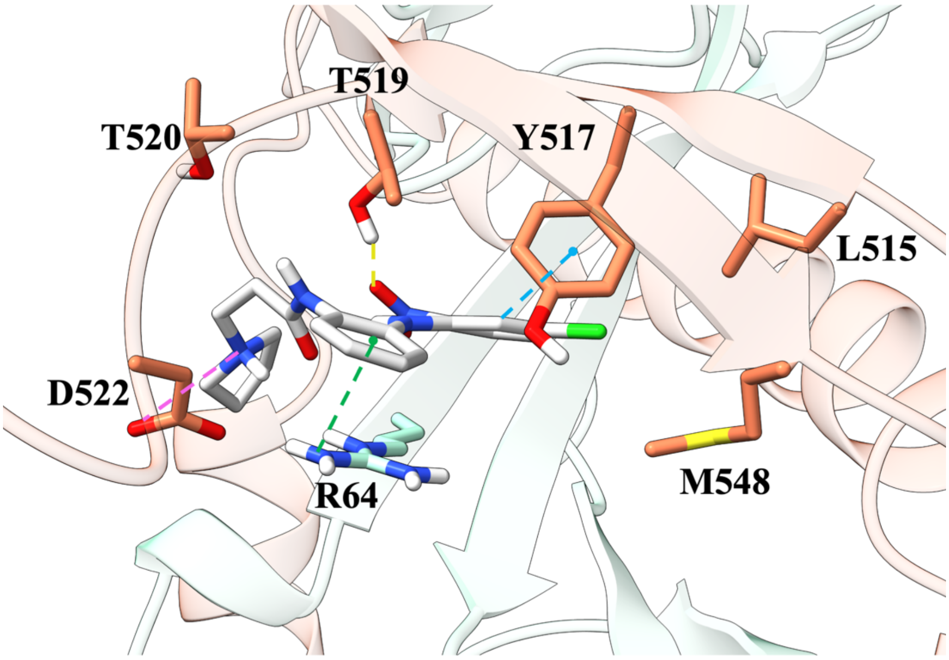

Figure S1. Representative binding mode of compound **17** (rendered in grey sticks) within the cavity, formed between GP1 (illustrated as a cyan cartoon) and GP2 (depicted as a salmon cartoon). Detailed close-up of the ligand binding pocket, highlighting essential residues within a 5Å proximity of the ligand. The yellow dashed line denotes hydrogen bond, while the pink dashed line indicates salt bridge, the blue dashed line represents π-π stacking and green dashed line shows π-cation interaction. The X-ray structure of the EBOV-GP in complex with toremifene (PDB ID: 5JQ7) was used as a starting point for molecular modeling studies.

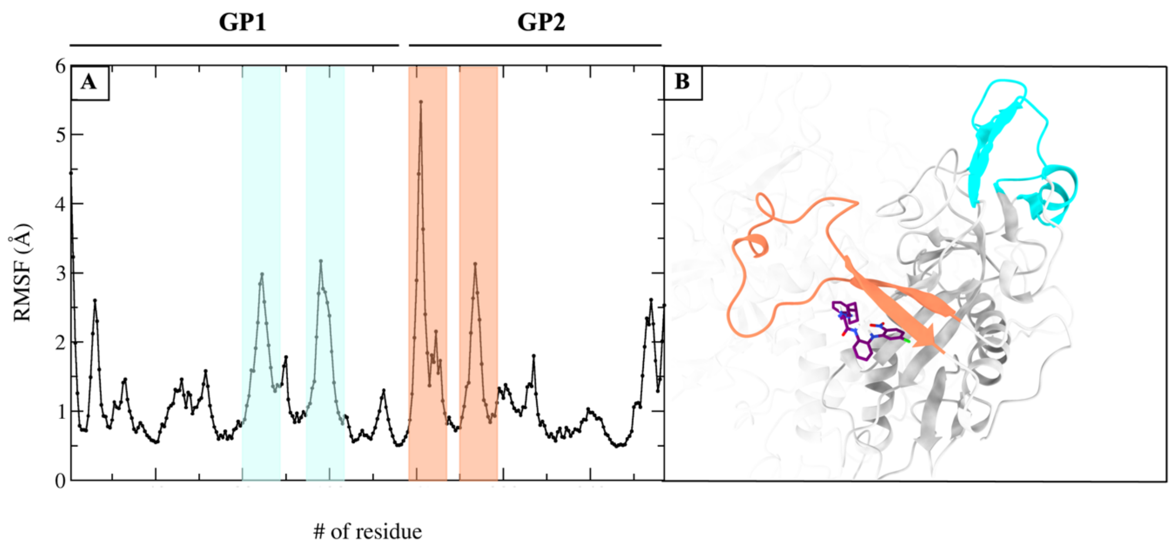

Figure S2. A) RMSF (Å) for the average of the three independent replicas of GP-**16** simulated system. The highlighted regions correspond to the fusion loop (in salmon) and receptor (NPC1) binding sites (in cyan). B) Residues forming the NPC1 binding surface and the fusion loop of the EBOV-GP are highlighted respectively in cyan and salmon. Compound **16** is shown in purple sticks.

Figure S3. Linear correlation between experimental and reported permeability of ten commercial drugs used for the experiment validation in the PAMPA assay.

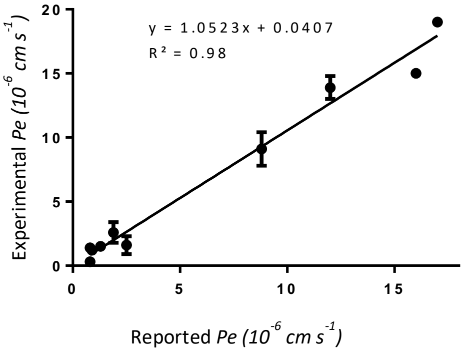

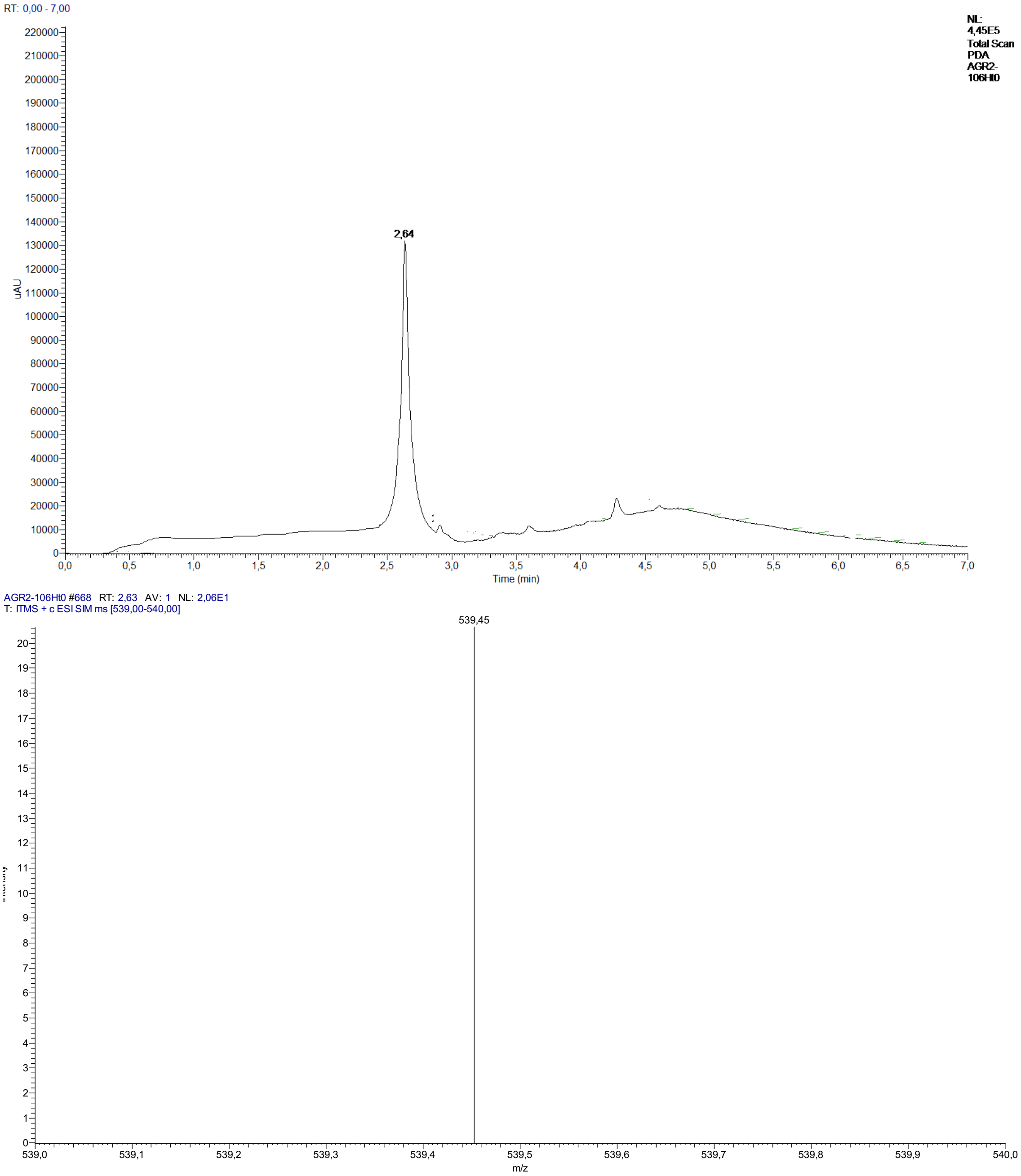

Figure S4. HPLC/MS chromatogram of compound **11**.

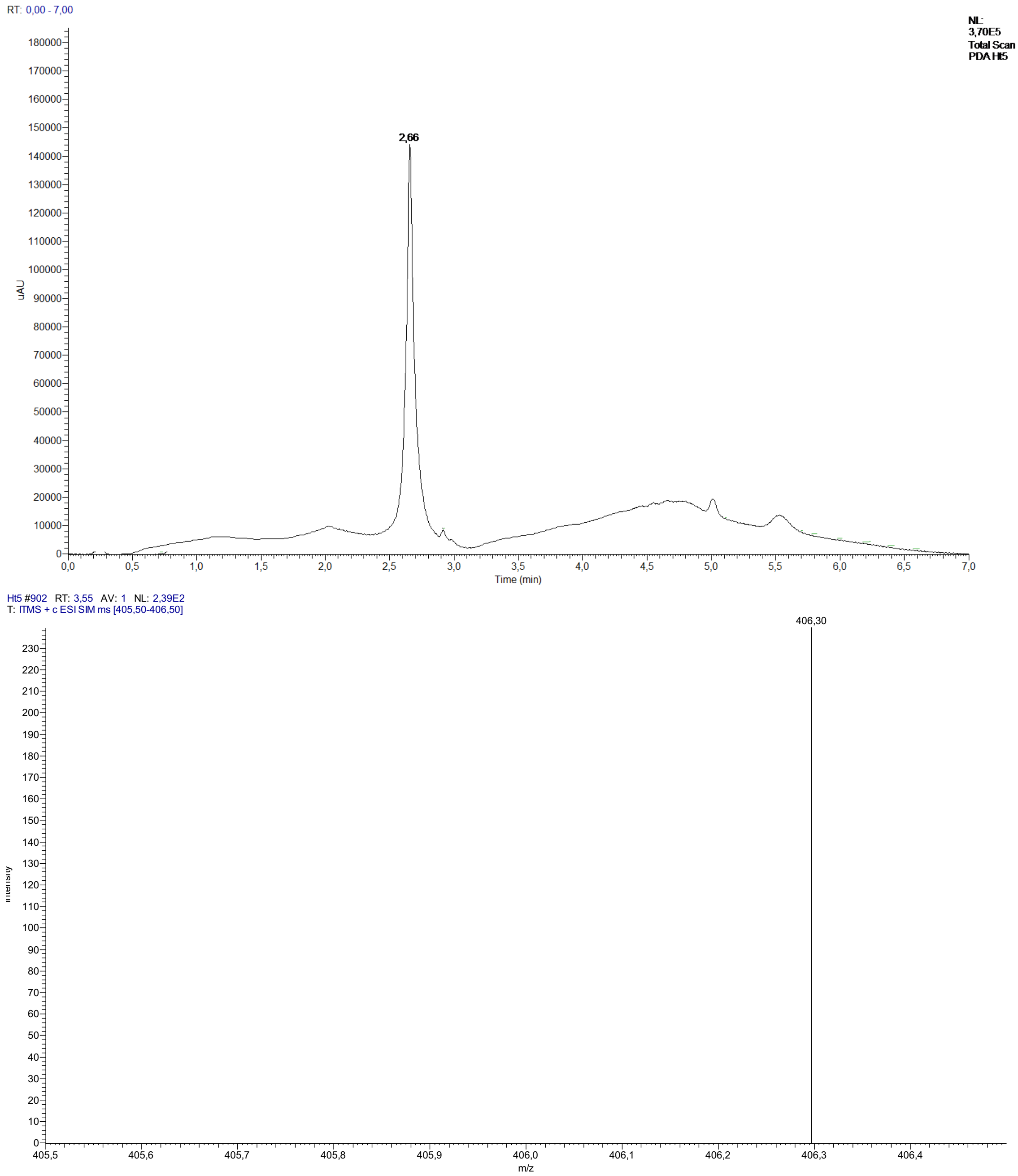

Figure S5. HPLC/MS chromatogram of compound **12**.

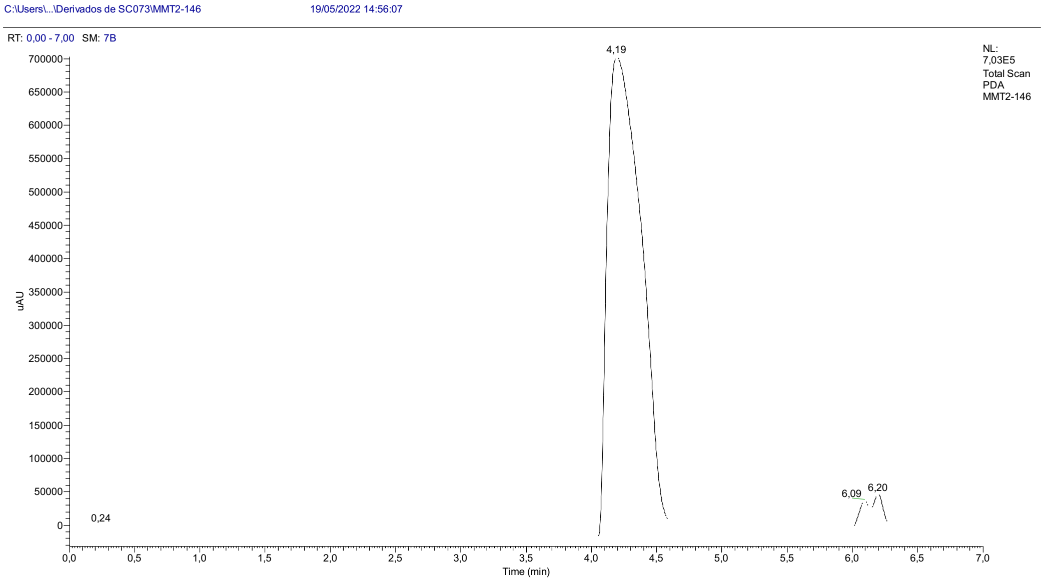

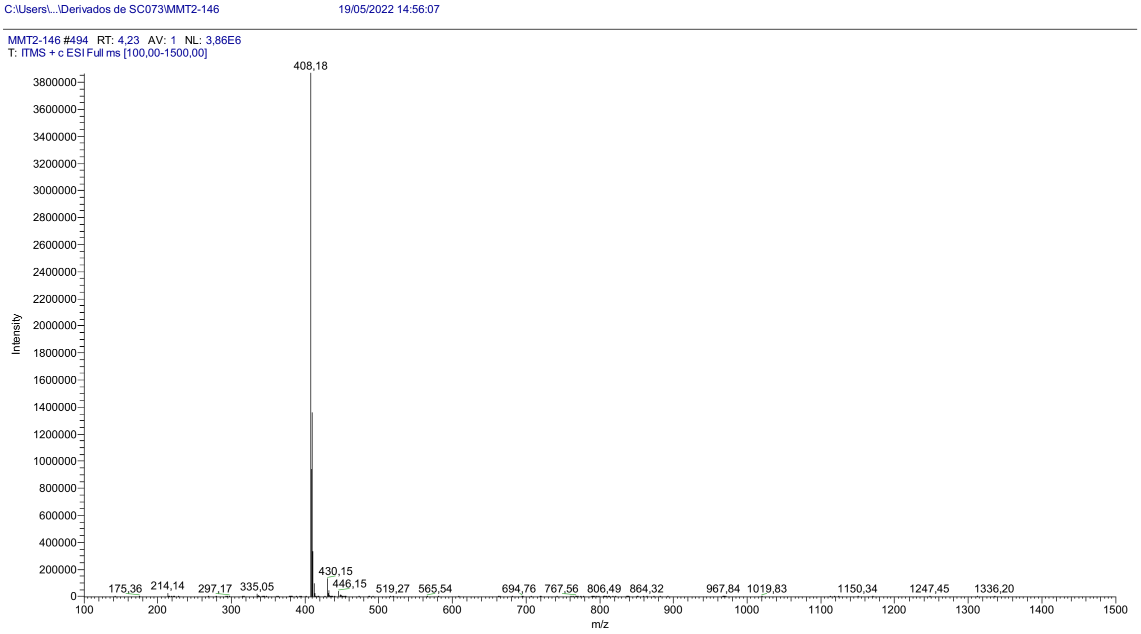

Figure S6. HPLC/MS chromatogram of compound **13**.

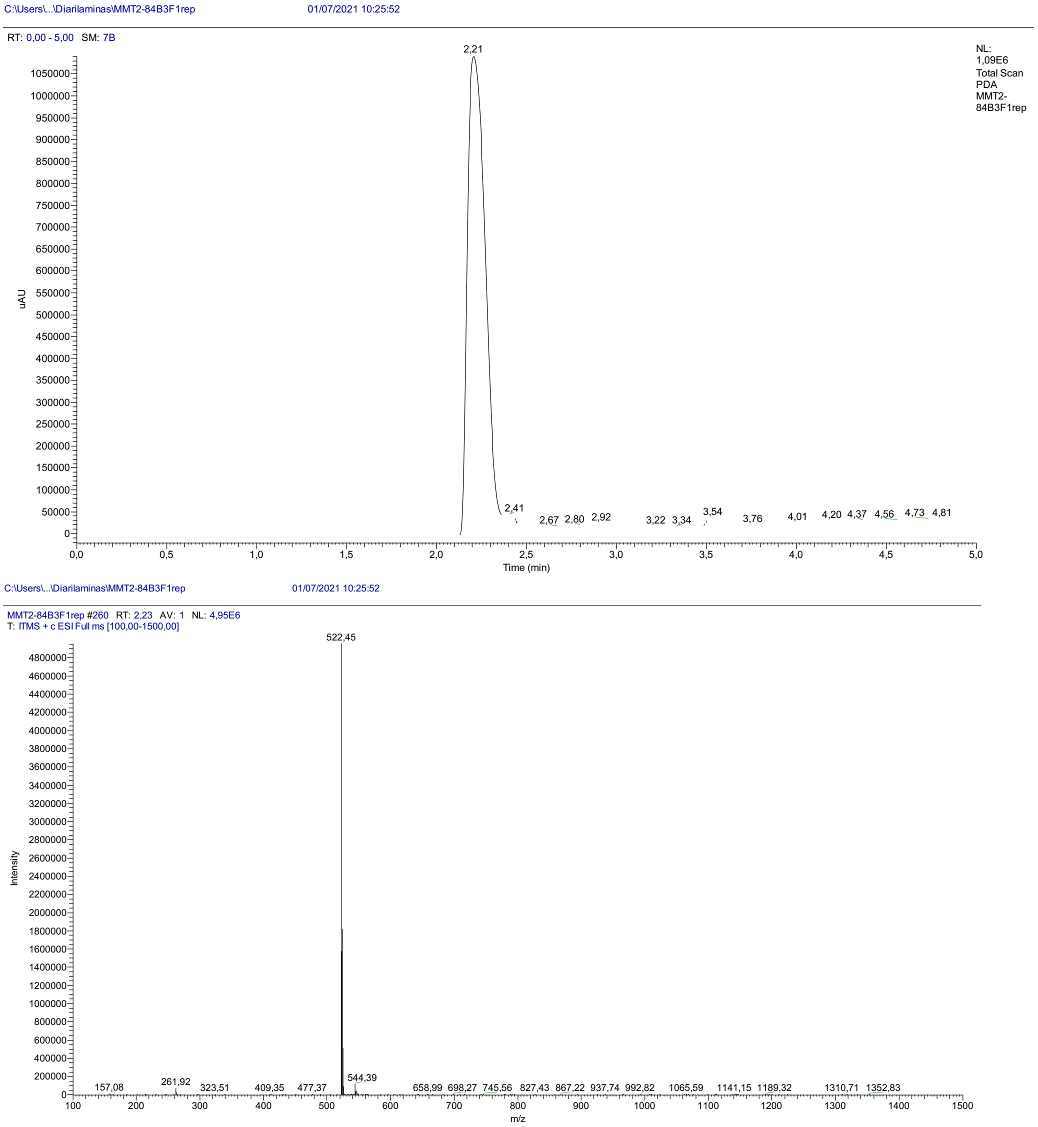

Figure S7. HPLC/MS chromatogram of compound **16**.

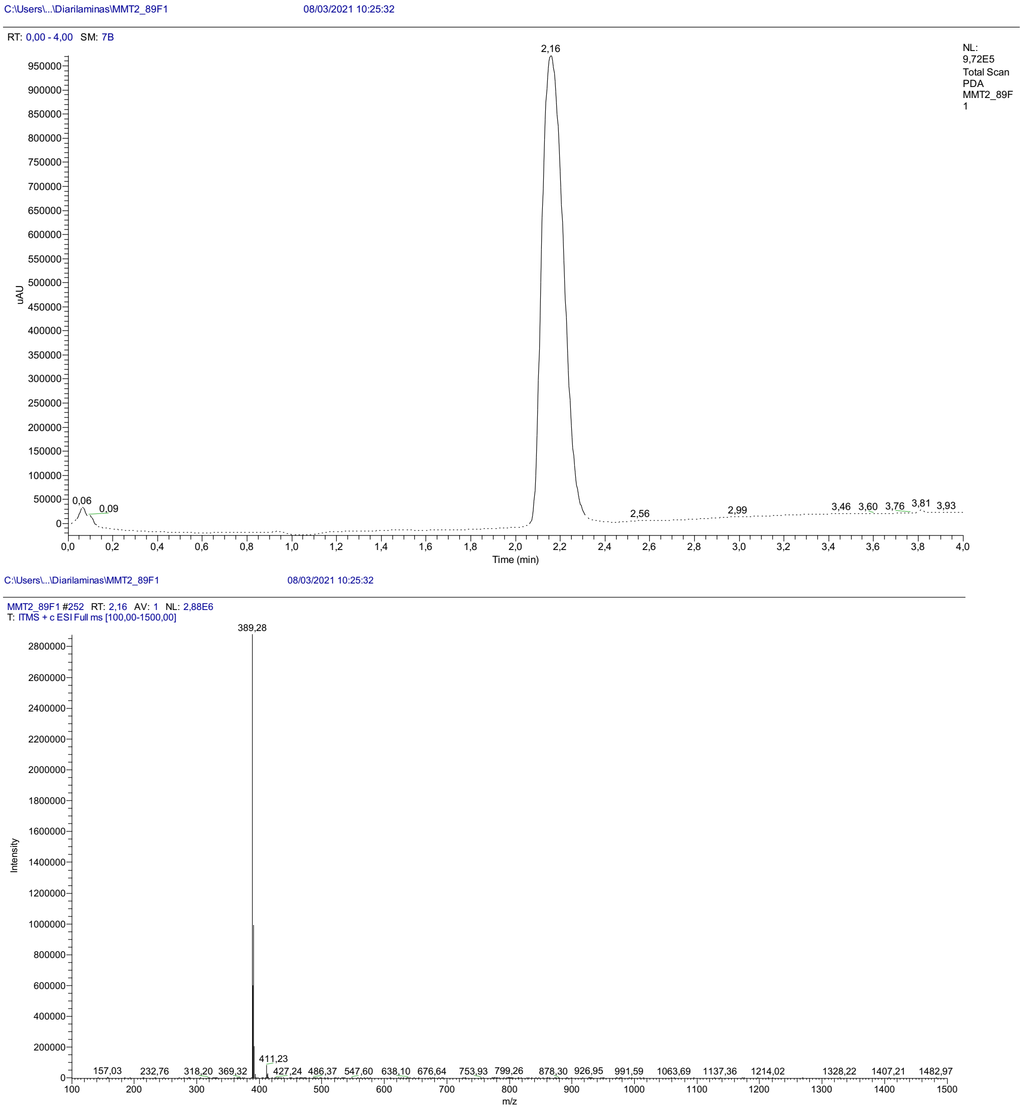

Figure S8. HPLC/MS chromatogram of compound **17**.

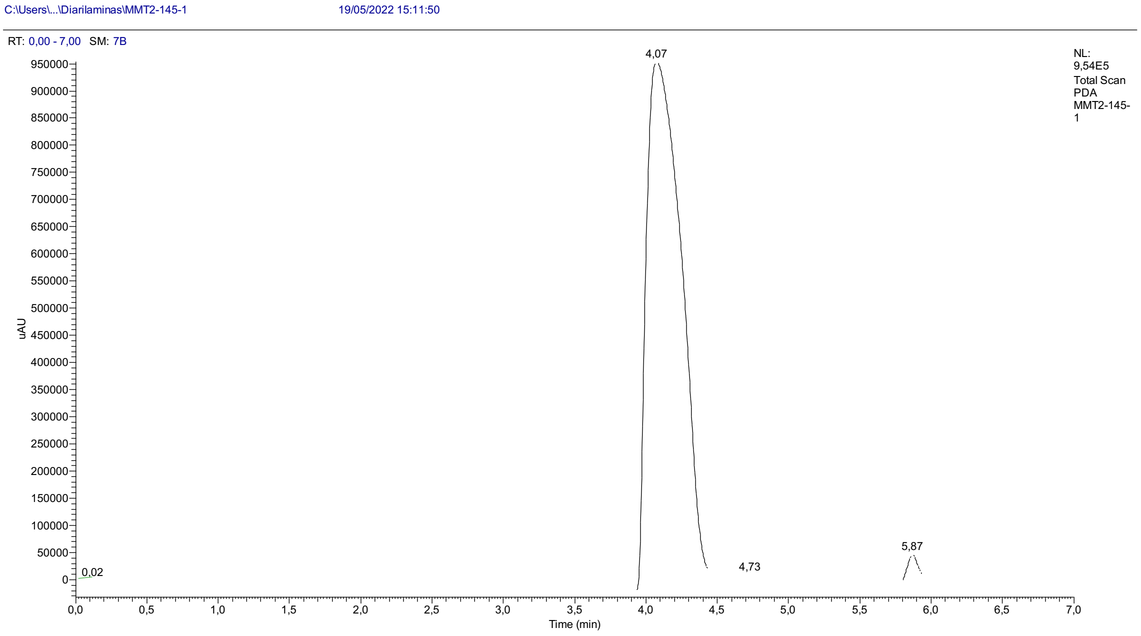

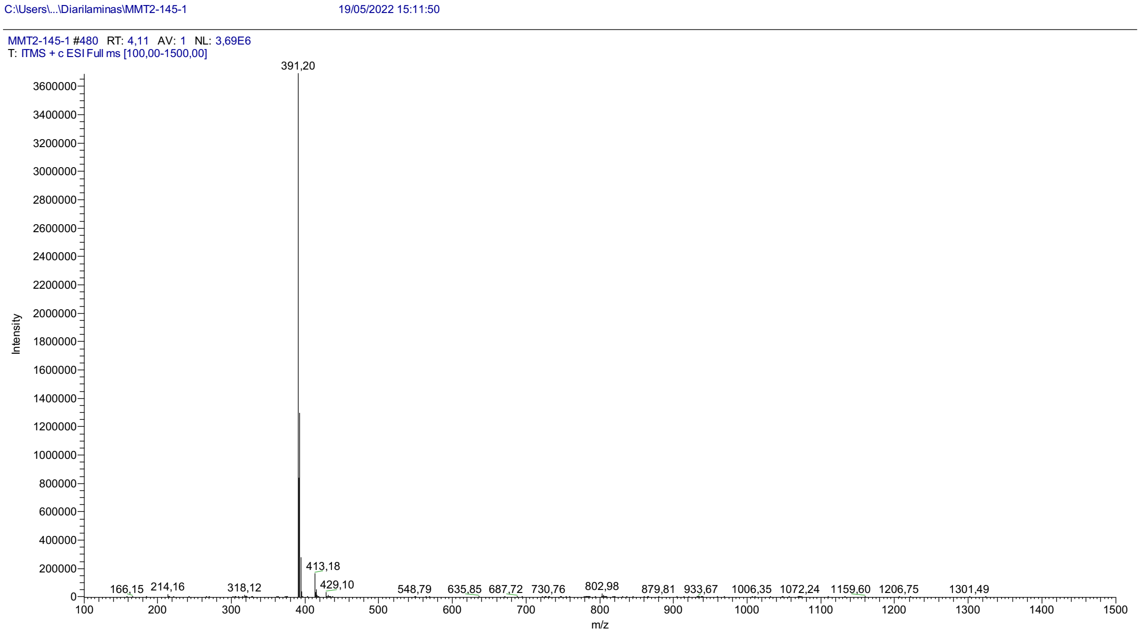

Figure S9. HPLC/MS chromatogram of compound **18**.

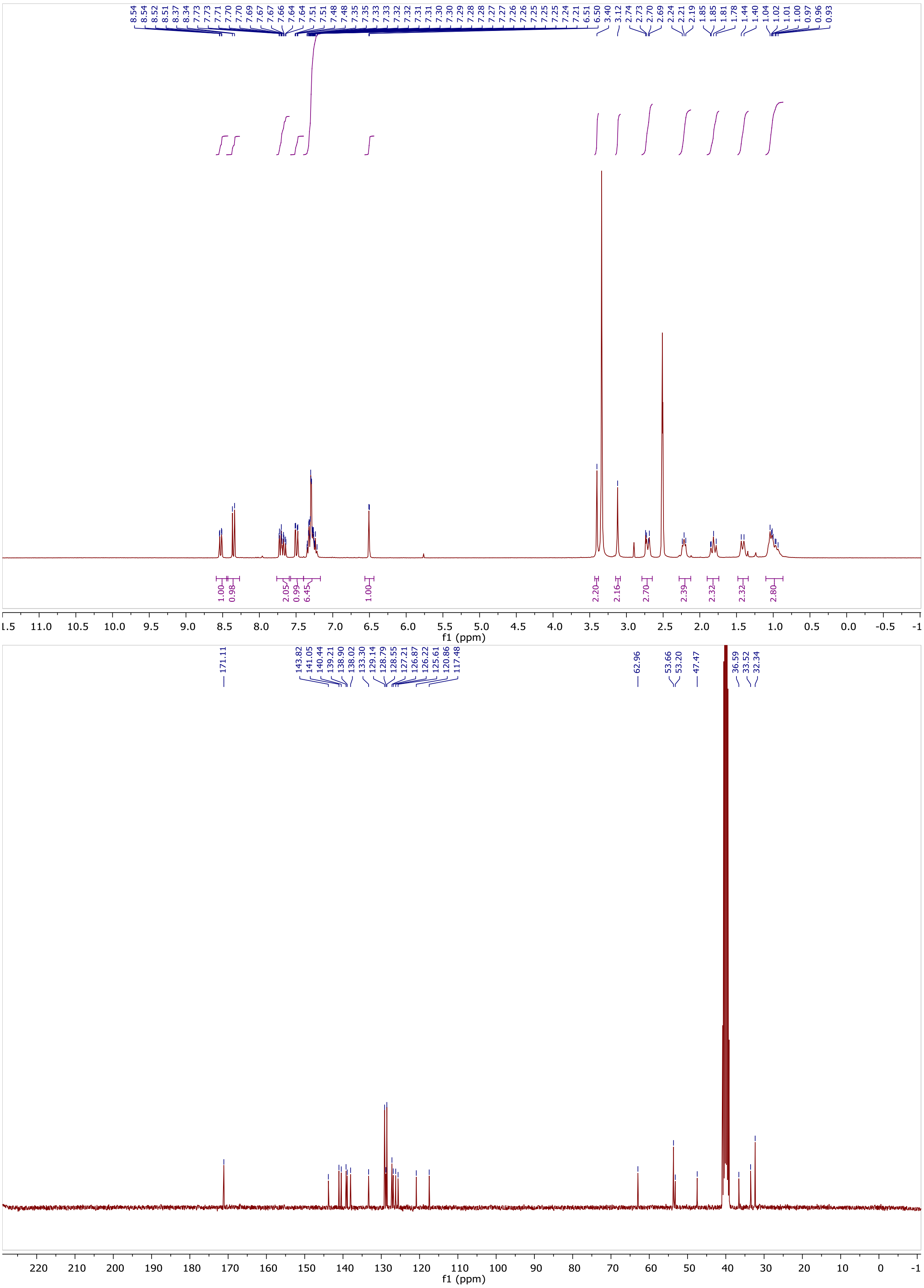

Figure S10. ^{1}H NMR and ^{13}C NMR spectra of compound **11**.

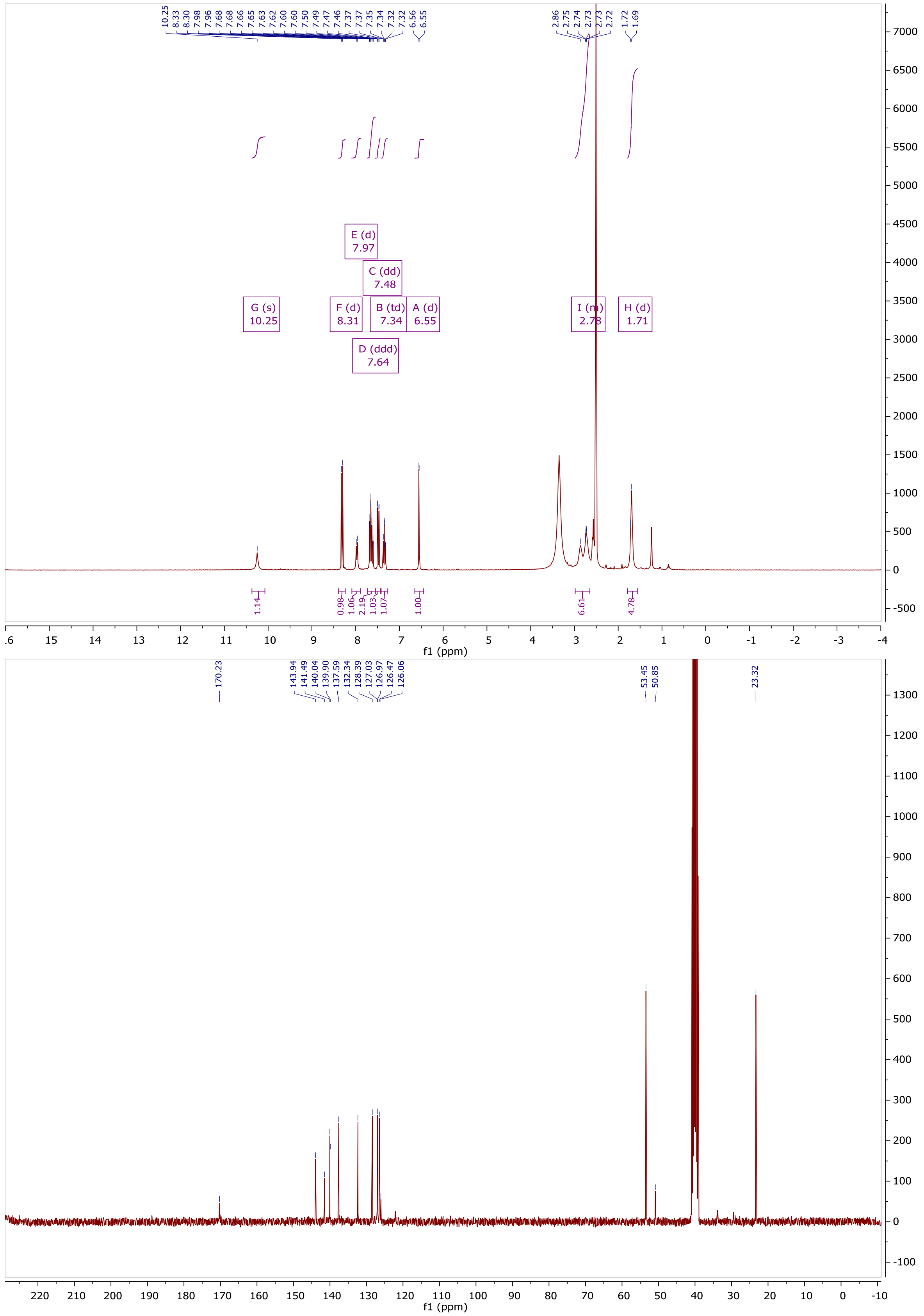

Figure S11. ^{1}H NMR and ^{13}C NMR spectra of compound **12**.

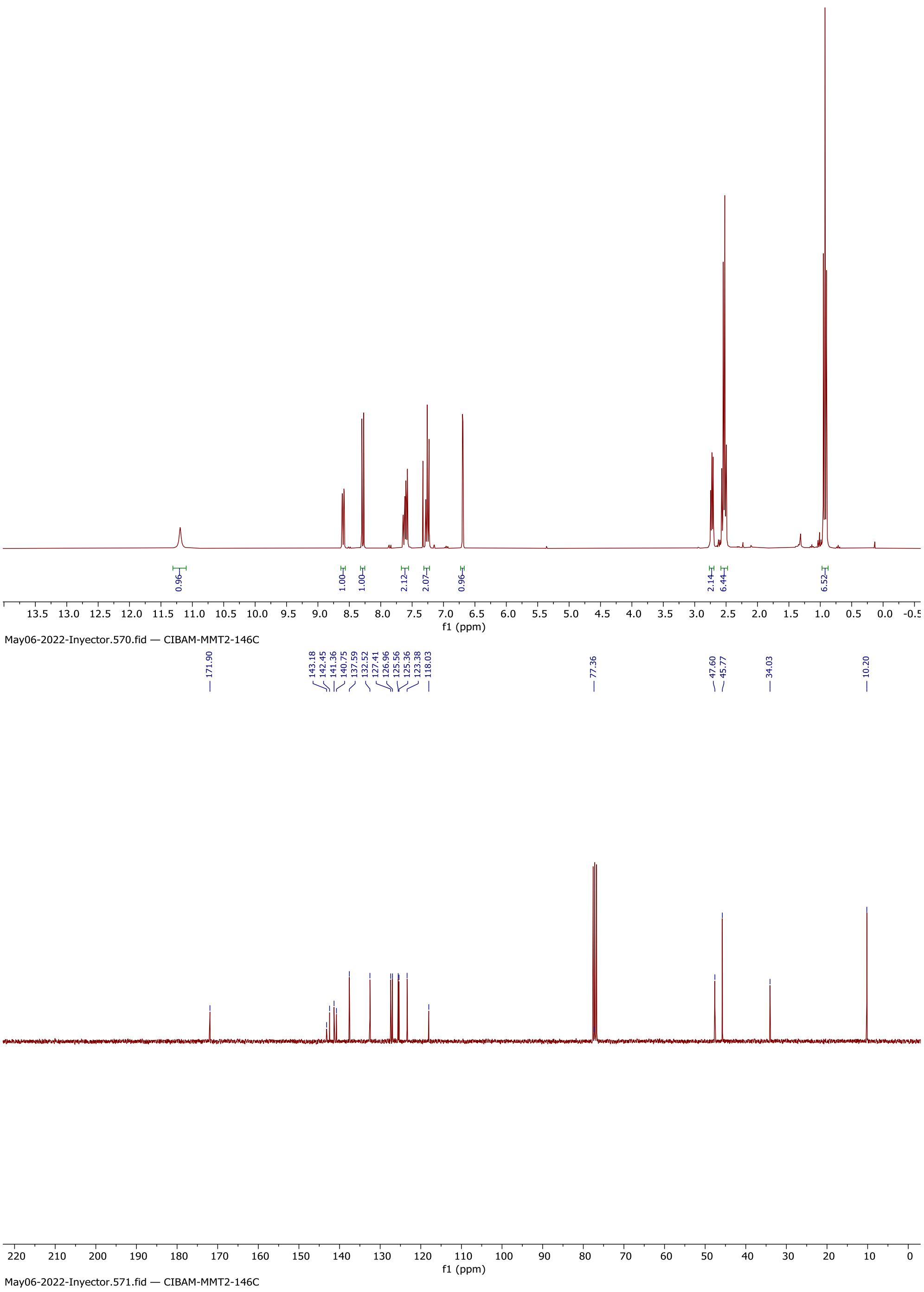

Figure S12. ^{1}H NMR and ^{13}C NMR spectra of compound **13**.

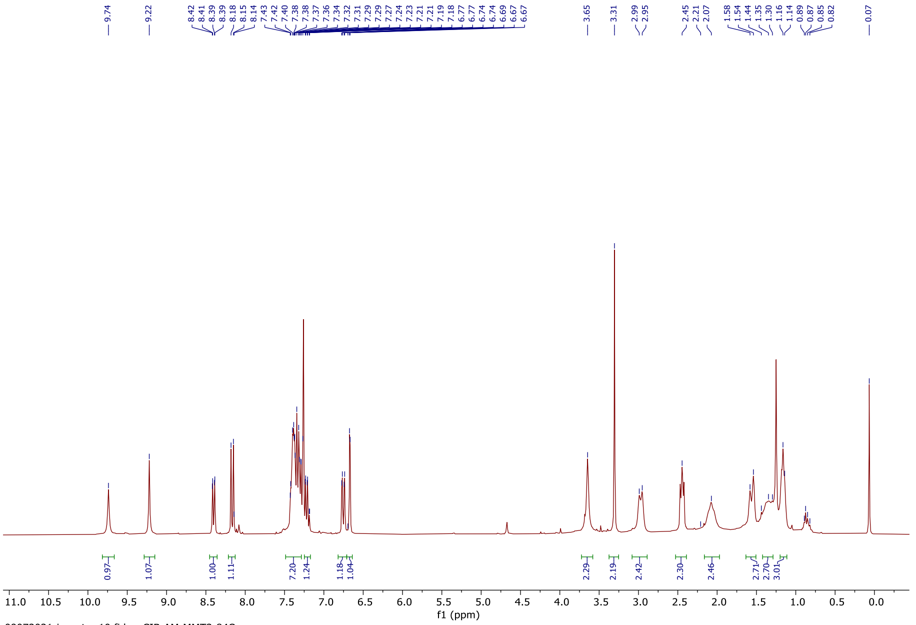

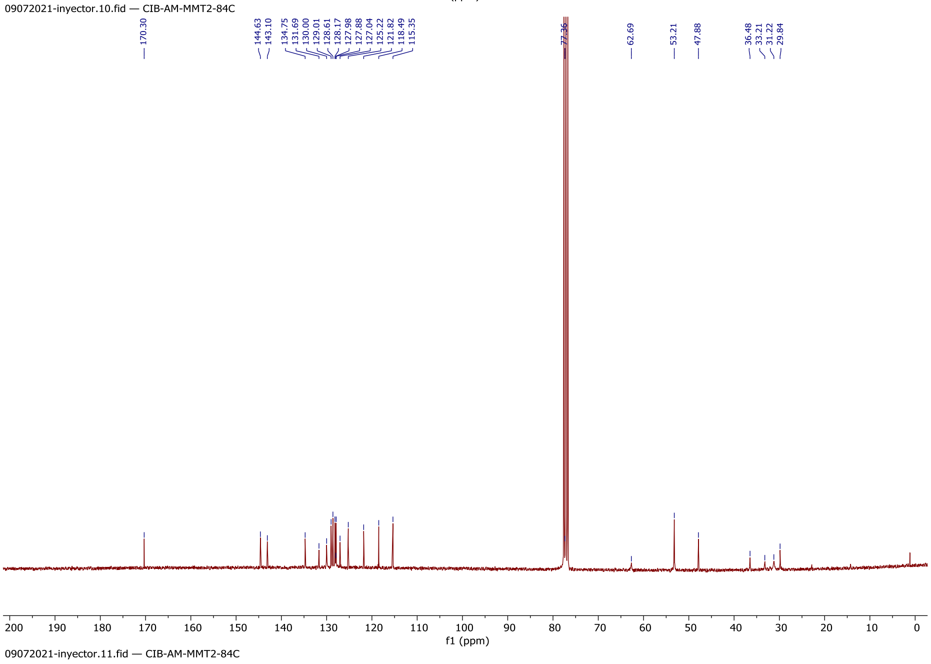

Figure S13. ^{1}H NMR and ^{13}C NMR spectra of compound **16**.

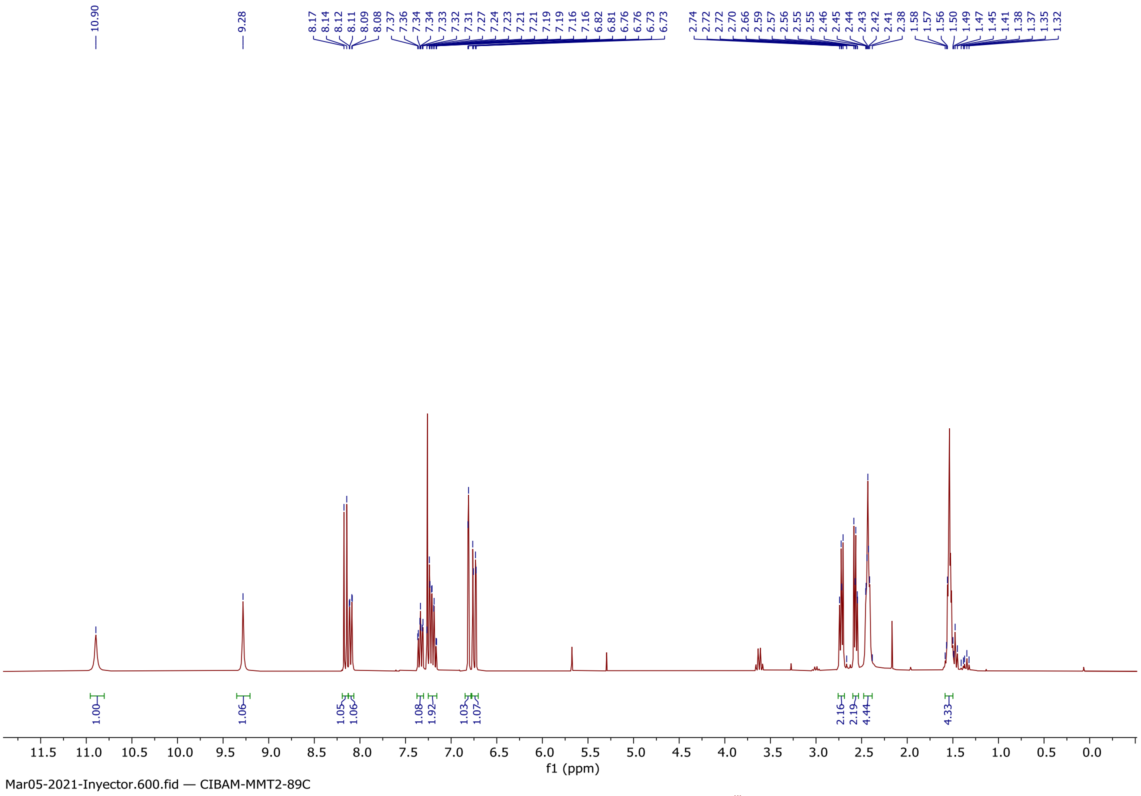

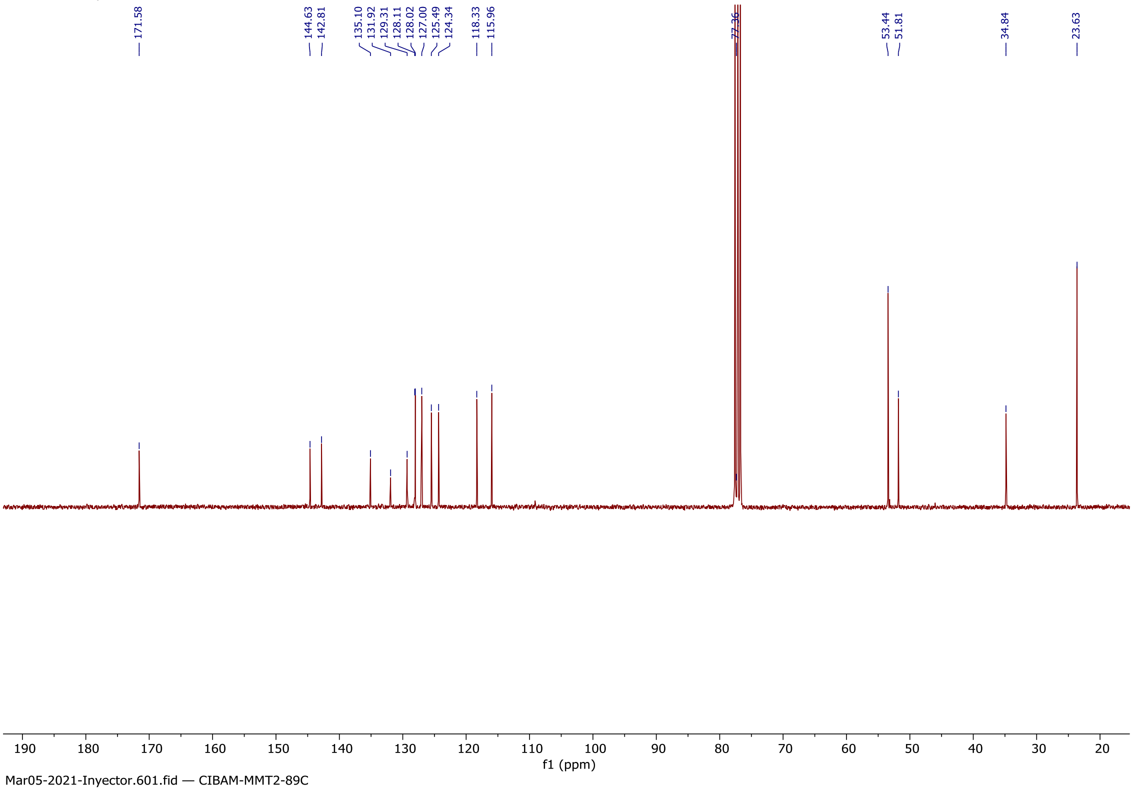

Figure S14. ^{1}H NMR and ^{13}C NMR spectra of compound **17**.

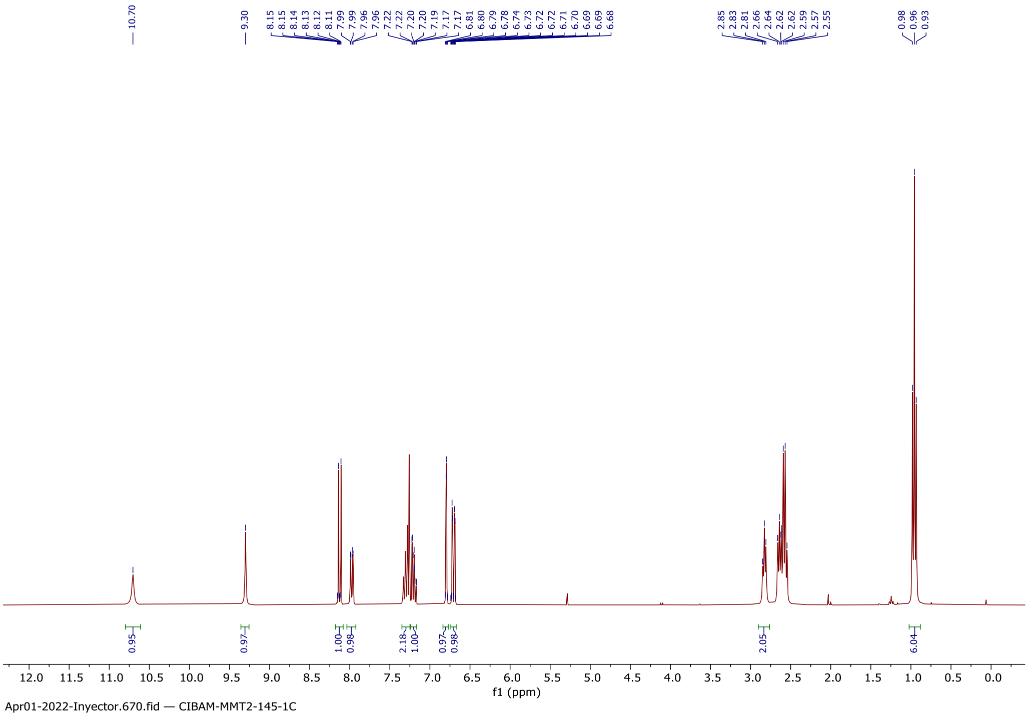

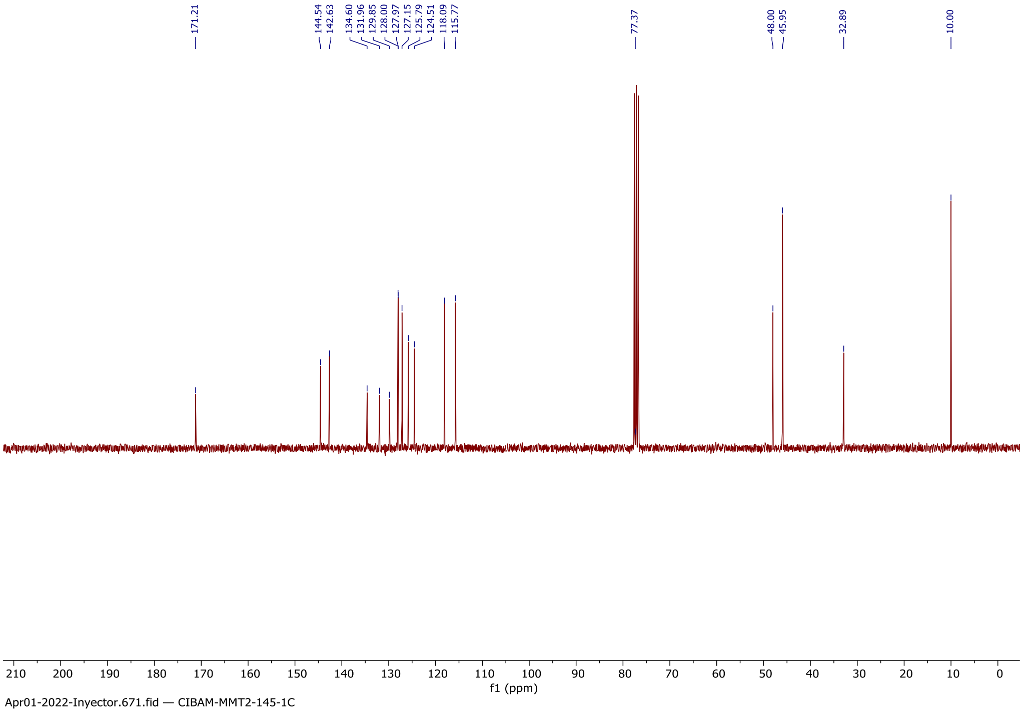

Figure S15. ^{1}H NMR and ^{13}C NMR spectra of compound **18**.
